# Bioproduction of ∼10 knt single-stranded DNA for constructing large DNA origami structures

**DOI:** 10.1016/j.mtbio.2026.103092

**Published:** 2026-04-14

**Authors:** Meiling Lu, Xiwei Wang, Baohong He, Jingyan Zhang, Youqing Cu, Jinjing Che, Nan Liu, Zengming Wang, Hui Zhang, Liang Xu, Xuili Gao, Aiping Zheng

**Affiliations:** a State Key Laboratory of Discovery and Utilization of Functional Components in Traditional Chinese Medicine & School of Pharmaceutical Sciences, Guizhou Medical University, Guiyang, 561113, China; b Academy of Military Medical Sciences, Beijing, 100850, China

## Abstract

*DNA origami structures based on M13 scaf*folds have limitations in terms of functional integration and size expansion. Using longer scaffold strands can substantially increase the number of programmable functional sites and size of DNA origami. Therefore, preparing long single-stranded DNA (ssDNA) and stably synthesizing large DNA origami structures are key challenges. Here, we established a highly efficient biosynthesis platform for long ssDNA (∼10 knt) based on a phage-phagemid production system, and used this ssDNA to prepare large DNA origami structures with stable structure and high yield. The yield of the long ssDNA was increased by approximately an order of magnitude by optimizing the synthesis parameters in shake flasks and bioreactors. We successfully constructed large triangular (161 nm side length) and rectangular (93 × 115 nm) DNA origami structures, which have approximately 100 more potential functionalization sites compared to traditional structures based on M13mp18. This work contributes to improve the scalable production of long ssDNA scaffolds and provides a practical foundation for constructing large DNA origami nanostructures.

## Graphical abstract

**Figure:**
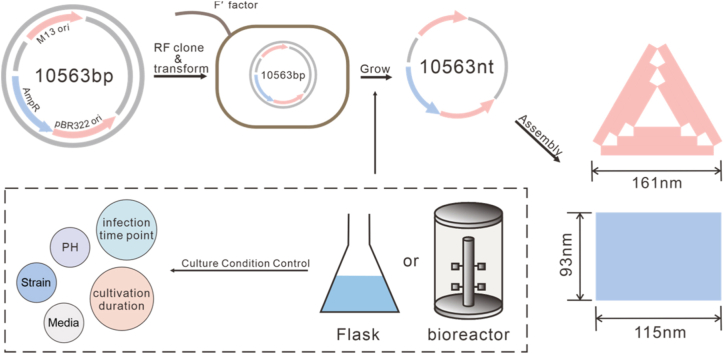

## 1 Introduction

Conventional DNA origami relying on the M13mp18 (7429 nt) scaffold presents challenges for extensive functional integration and adjustment of structural dimensions [1,2], which restricts the application in frontier areas such as protocell construction [3], DNA nanopore-based channel regulation [4,5], and drug delivery [6,7]. Employing longer single-stranded DNA (ssDNA) scaffolds (>7429 nt) to assemble larger DNA origami architectures offers a promising strategy to substantially increase the number of programmable functional sites and structural dimensions. At present, commercially available long ssDNA are primarily available in lengths of 4844 nt, 7249 nt, 7559 nt, 7560 nt, 8064 nt, and 8634 nt. Therefore, the efficient and scalable production of long single-stranded DNA (ssDNA) exceeding 10,000 nt is a major challenge, which restricts the size expansion and integration capabilities of DNA origami. The synthesis strategy via phosphoramidite chemistry is limited to lengths of 300-400 nt due to incorporation errors and depurination [8]. To obtain long ssDNA, a variety of alternative strategies have been developed. A symmetric polymerase chain reaction (PCR) using double-stranded DNA (dsDNA) as a template [9], as well as the strategies combining the Cas9 nickase with T7 exonuclease, can generate ssDNA up to approximately 15,000 nt in length [10]. In addition, long single-stranded DNA can be generated using differentially modified primers, such as lambda exonuclease digestion with phosphorylated primers or streptavidin based separation using biotinylated primers [11,12]. However, these techniques typically yield <1 μg of ssDNA per 50 μL reaction, making the production of milligram quantities costly and inefficient due to the extensive labor and high reagent consumption [13]. Therefore, this underscores the necessity for more scalable and economical ssDNA production methods.

Bioproduction of ssDNA in *Escherichia coli* (*E. coli*) offers a scalable alternative. In this approach, the target genome is introduced into *E. coli* via phage-specific infection or transformation with a recombinant phagemid [14,15]. The intracellular rolling-circle replication machinery then generates ssDNA, which is subsequently harvested and used as a scaffold for the construction of DNA origami nanostructures [[16], [17], [18]]. At present, the bioproduction of ssDNA in *E. coli* has achieved relatively high yields within a moderate length range. For example, ssDNA with a length of 7249 nt can be produced at yields of approximately 590 mg/L [19,20]. While shorter ssDNA molecules (e.g., 2800 nt and 3200 nt) can also reach yields of around 141 mg/L [21]. However, when the ssDNA length further exceeds 10 knt, the biosynthetic yield decreases sharply, with the production of ∼10 knt ssDNA approximately 1 mg/L [22]. This pronounced length-dependent decline in production efficiency severely limits the application of ultralong ssDNA in the construction of large DNA origami. Moreover, the use of longer ssDNA scaffolds markedly increases the propensity for intramolecular secondary structure formation, thereby adversely affecting folding efficiency and yield of large DNA origami structures [23].

**Fig. 1:**
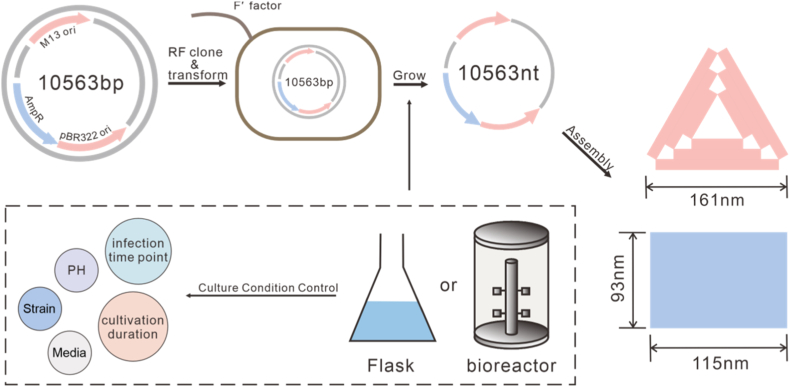
Schematic illustration of the biosynthesis of long ssDNA. The PCR-amplified dsDNA fragments were first assembled into recombinant phagemids using Gibson assembly and subsequently transformed into *E. coli* for amplification. This process was carried out in both shake-flask cultures and bioreactor systems, with systematic optimization of cultivation conditions to markedly enhance phage particle production. Finally, the resulting ssDNA was harvested and purified, and subsequently employed as a scaffold strand for the assembly of large-scale DNA origami nanostructures.

In this study, we achieved the biosynthesis of approximately 10 knt ssDNA using a helper phage–mediated phagemid system in *E*. *coli.* The biosynthetic conditions for ssDNA production were first systematically optimized in shake-flask cultures to improve yield and stability. Subsequently, the optimized process parameters were scaled up and implemented in a batch fermenter process (Fig. 1) using a bioreactor, resulting in a substantial enhancement of ssDNA production. Then, we employed the ssDNA as scaffold strands for constructing large DNA origami structures. Given that the long scaffold, which promotes the formation of intramolecular secondary structures and thereby reducing correct folding efficiency, we optimized the ratio of scaffold strands to staple strands and the scaffold concentration to improve both the folding efficiency and overall yield of large DNA origami structures.

## 2 Materials and methods

**Recombinant phagemid construction.** Four DNA fragments with lengths of 5648 bp, 1782 bp, 2611 bp, and 660 bp were individually amplified by PCR. These fragments were rationally designed to contain mutually overlapping homologous ends and were subsequently seamlessly assembled using the Gibson assembly method, resulting in the successful construction of the full-length recombinant phagemid.

Recombinant phagemids were then transformed into chemically competent *E. coli* for further amplification and subcloning analysis. 100 μL of competent cells were transformed with 100 ng of recombinant phagemids. Cells were incubated on ice for 30 min, heat shocked at 42 °C for 45 s, and then put on ice. Pre-warmed LB media was added and the cell culture was shaken at 37 °C for 1 h 100 μL of cells were plated evenly across a LB media plate made with 100 μg/mL ampicillin. The recombinant phagemids were verified bygene sequencing, and the ones with correct sequences were transformed into *E. coli* XL1-Blue (TSINGKE)、DH5a (Thermo Fisher)、JM109 (TSINGKE) cells for ssDNA production.

**Transmission Electron Microscopy (TEM) Characterization of Phagemid Particles.** The culture supernatant obtained after centrifugation was deposited onto a copper grid and allowed to stand briefly to facilitate solvent evaporation. The samples were subsequently negatively stained with 1% (w/v) uranyl acetate for 1 min. TEM imaging was performed using a Hitachi HT7800 transmission electron microscope operated at an accelerating voltage of 80 kV.

**Exonuclease I Digestion Assay.** For the exonuclease digestion assay, 10,563 nt ssDNA, M13mp18 ssDNA, and linear ssDNA (200 ng each) were incubated with 1.75 μL Exonuclease I (New England BioLabs) in a reaction volume of 50 μL. The reaction mixture was processed in a PCR thermocycler, with incubation at 37 °C for 15 min, followed by 80 °C for 15 min to inactivate the enzyme.

Linear ssDNA was prepared by hybridizing a short complementary oligonucleotide to the target region to introduce an *Eco*RI restriction site, followed by restriction enzyme digestion to generate linear single-stranded DNA.

**ssDNA production in shaker flask.** We optimized the shaker flask production conditions of ssDNA to enhance the yield of long ssDNA. *E. coli* cells harboring the correctly assembled phagemid were grown to an OD_600_ of 0.5, after which 1 mL of the culture was inoculated into 250 mL of medium supplemented with 100 μg/mL ampicillin. After cultivation for the indicated period, 50 μL of M13K07 (New England BioLabs) helper phage was added to initiate infection. Based on this setup, key parameters such as the timing of helper phage infection, cultivation duration, culture medium, host strain, and cultivation pH were systematically evaluated and optimized. pH optimization was achieved by precise adjustment of the culture pH through the addition of 25% ammonium hydroxide.

The cultures were spun down at 5,000 rpm for 15 min, after which the supernatant was removed to a fresh tube and spun at 3000×*g* for an additional 15 min. Phage particles were precipitated by adding 1/100 volume of Buffer MP, followed by vortexing and incubation at room temperature for at least 2 min. The clarified phage-containing supernatant was then applied to QIAprep spin columns (Qiagen #27104). The columns were centrifuged for 15 s at 8000 rpm, and the flow-through was discarded. This loading and centrifugation step was repeated until the entire sample had been processed. Bound phage particles were lysed by adding Buffer PB and centrifuging for 15 s at 6000×*g*, followed by a second addition of Buffer PB, incubation for 1min at room temperature, and centrifugation under the same conditions to ensure complete lysis and DNA binding. To remove contaminants, the column was washed with 0.7 mL of Buffer PE and centrifuged for 15 s at 6000×*g*. After discarding the wash flow-through, the column was centrifuged again for 15 s to eliminate residual ethanol. The purified ssDNA was eluted by adding 100 μL of Buffer EB to the center of the membrane, incubating for 10min at room temperature, and centrifuging for 30 s at 8000 rpm. For enhanced recovery, the elution buffer could be preheated to 50 °C. The purified eluate DNA concentration was determined by A280 absorbance from a NanoDrop LITE (Thermo Fisher) for each time point and condition tested, and ran on a 1% agarose gel in 1 × Tris-Acetate-EDTA (TAE) stained with SolarRed (solarbio) for visualization of the product. The ssDNA purity was judged by ImageJ intensity analysis and the amount of ssDNA from the time point or condition was adjusted by this purity multiplied by the total amount of DNA found from A280 absorbance.

**ssDNA production in bioreactor.** Milligram-scale production of synthetic phage particles was carried out in a stirred-tank bioreactor (TMAXTREE Tmax Bio-3L) with a working volume of 1 L. XL1-Blue cells harboring the correctly assembled phagemid were grown to an OD_600_ of 0.5, and 4 mL of this culture was inoculated into 2 × YT medium supplemented with 100 μg/mL ampicillin. After 30 min of cultivation, 200 μL of M13K07 helper phage was added. During cultivation, the temperature was maintained at 37 °C, and the pH was controlled at 6.7 using 25% NH_4_OH. Dissolved oxygen was maintained at 25% air saturation by supplying sterile air at a flow rate of 2 vvm, with system pressure up to 2 bar and a maximum stirrer speed of 1000 r.p.m. Time points were taken approximately 2 h and samples were processed as above for the shaker flask. The optical density of the cultures at 600 nm (OD_600_) was measured using a microplate reader (Thermo Scientific™ Varioskan™ LUX), and cell numbers were calculated based on the following conversion: 1 OD_600_ = 1 × 10^9^ cells per mL culture. The purified eluate DNA concentration was determined by A280 absorbance from a NanoDrop LITE (Thermo Fisher) for each time point.

**Origami Designs.** Triangle origami structure and rectangleorigami structure was designed using CaDNAno software on the square lattice.

**Origami Assembly.** Samples (50 μL in total volume) were prepared by mixing the scaffold strand with corresponding staple strands in the presence of 1xTAE/Mg^2+^ buffer (40 mM Tris-Acetate, pH 8.0, 1 mM EDTA, and 12.5 mM Mg^2+^). Thermal anneals consisted of heating to 85 °C, holding for 3 min, cooling to 20°Cover 18 h, and a quick drop to 4 °C in a Themo thermal cycler.

**Liquid-phase AFM imaging.** 3 μL of 1 mM NiCl solution was first deposited on a piece of freshly cleave mica for 2 min. After that, 3 μL of DNA origami samples were deposited onto this mica surfacefor 2 min. At last, 25 μL 1xTAE/Mg^2+^ buffer was added onto the mica. Imaging was performed on MultiMode V AFM (Bruker) in ScanAsyst-Liquid mode, using SNL-10tips (Bruker).

**Air-phase AFM imaging.** 3 μL of DNA origami samples were deposited onto the mica surface for 3 min. The substrate was then washed with distilled water to remove any unabsorbed samples and dried with high-purity nitrogen. Imaging was performed using a MultiMode V AFM (Bruker) in TappingMode, with BRUKER RTESP-300 probes and a Drive Amplitude of 45 mV.

## 3 Results

### 3.1 Biosynthesis of 10,563 nt ssDNA

**Fig. 2:**
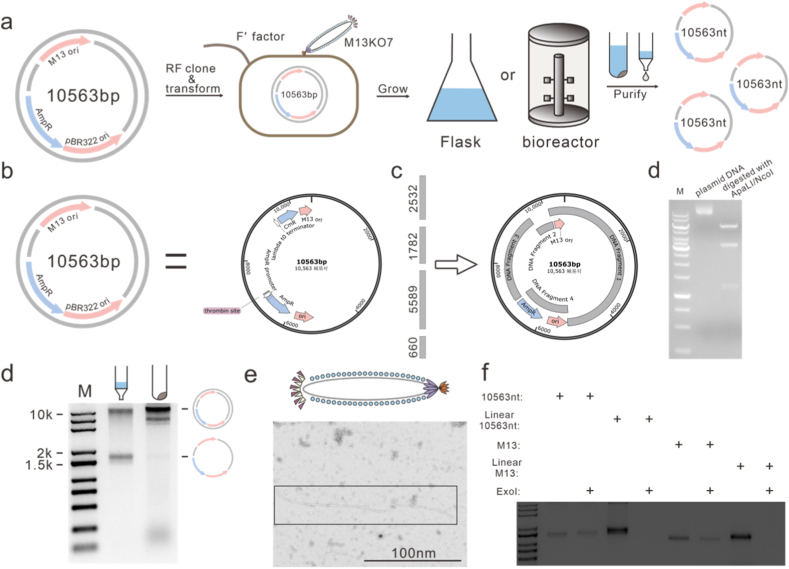
Scalable bacteriophage production of 10,563 nt ssDNA. (a) Schematic representation and map of the 10,563 bp recombinant phagemid. (b) Four DNA fragments (660 bp, 5648 bp, 1782 bp, and 2611 bp) were synthesized by PCR and assembled using Gibson assembly to generate the 10,653 bp recombinant phagemid. (c) *Apa*LI/*Nco*I digestion of the recombinant phagemid yielded bands matching the SnapGene-predicted fragment sizes, confirming successful construction. (d) DNA purification from the bacterial pellet and the clarified media showed mostly pure cssDNA in the media and cssDNA and dsDNA phagemid in the bacterial pellet. (e) Phage particles from clarified media were visualized by TEM. (f) Stability from ExoI degradation after 30 min coincubation indicates the ssDNA is circular.

The biosynthesis of ssDNA was achieved through a helper phage–mediated phagemid system, in which the helper phage supplied the essential capsid proteins and facilitated rolling-circle replication of the phagemid to generate circular ssDNA. Consequently, the length of the resulting ssDNA is directly determined by the sequence length of the phagemid. The schematic illustration of the ssDNA biosynthesis workflow is shown in Fig. 2a. The recombinant phagemid with 10,563 bp was introduced into *E. coli* host cells carrying the F′ factor, followed by cultivation in shake flasks or bioreactors. Then, the resulting culture was used for the extraction and purification of ssDNA. During purification, phage particles in the culture were captured on a silica membrane column and lysed directly on the column, allowing the released ssDNA to bind to the silica matrix while contaminants were removed during the washing steps. Because the ssDNA remains protected within phage particles during the initial purification process, this approach helps preserve the structural integrity of the long ssDNA scaffold.

Firstly, we constructed the recombinant phagemid with a total length of 10,563 bp (Table S1), which had been previously reported by Chen et al. [22]. The recombinant phagemid contained a high-copy-number pBR322 origin of replication to regulate its replication in *E. coli*, an M13-derived origin of replication to drive rolling-circle replication, and an ampicillin resistance gene for selection (Fig. 2b). The primer sequences were listed in Table S2. We generated four DNA fragments (5589 bp, 1782 bp, 2532 bp, and 660 bp) through PCR amplification, and then assembled these fragments using Gibson assembly to obtain the full-length recombinant phage plasmid (Fig. 2c). The fragments were amplified from the *E. coli* genome, λ DNA, and the pQE-80 L and pET-28a vectors to avoid introducing extensive repetitive sequences. M13 phage replication origins were designed with approximately 30 bp overlaps with adjacent DNA fragments to facilitate Gibson assembly. Restriction digestion was simulated in SnapGene using *Apa*LI and *Nco*I, yielding three predicted fragments of 1246 bp, 3284 bp, and 6033 bp (Fig. S1). In the wet experiment, the agarose gel electrophoresis image showed three bands, consistent with the expected results (Fig. 2d). To further validate the construction of recombinant phagemid, sequencing was performed. The obtained sequence was identical to the designed sequence (Supplementary Data 1). Then, the recombinant phagemid was introduced into chemically competent *E. coli* and selected on ampicillin-containing plates, yielding colonies with characteristic morphology (Fig. S2). The sequencing result of the monoclonal colonies was completely consistent with the gene sequence of the recombinant phagemid, indicating that the 10,563 bp recombinant phage plasmid was successfully introduced into *E. coli*.

Next, we synthesized 10,563 nt ssDNA in the shake flask. The *E. coli* cells were incubated for a total of 18 h in LB medium, with infection by the M13KO7 helper phage occurring 1 h after inoculation. To identify the main existing form of ssDNA, either in *E. coli* cells or phagemids, the culture was subjected to centrifugation. Subsequently, ssDNA extraction and purification were separately performed on the supernatant and the cell pellet. Based on their difference in density, the relatively lighter phagemid particles were primarily enriched in the supernatant, whereas the heavier *E. coli* cells were collected in the pellet. As shown in Fig. 2d and S3, the ssDNA band corresponding to 10,563 nt migrated above that of M13mp18 ssDNA (7249 nt), indicating that ssDNA was predominantly enriched in the culture supernatant, with only a small fraction detected in the cellular pellet. In addition, the band observed at approximately 10 kbp likely corresponds to phagemid, whereas the slightly lower-molecular-weight band may originate from the replicative double-stranded (RF) form of the M13KO7 helper phage. These results suggest that ssDNA is primarily released and recovered in a form encapsulated within phagemid particles, and that efficient ssDNA biosynthesis is, to some extent, dependent on the stable production of phagemid particles. Furthermore, transmission electron microscopy (TEM) characterization of phage particles in the supernatant revealed intact filamentous phage with normal morphology and an average length of approximately 200 nm (Fig. 2e; see Fig. S4 for the uncropped image), providing further evidence that ssDNA was effectively packaged within phage particles. In *E*. *coli*, ssDNA generated through a helper phage–mediated rolling-circle replication of the phagemid is produced in a circular form. To verify the circular topology of the obtained ssDNA, the samples were incubated with exonuclease I (ExoI); no detectable degradation was observed after 30 min (Fig. 2f), indicating that the ssDNA existed predominantly in a circular configuration, consistent with the rolling-circle replication mechanism. Collectively, these results confirm the successful biosynthesis of ssDNA with a length of 10,563 nt. Using this method, approximately 0.7 mg of ssDNA was obtained per liter of culture, with ImageJ analysis of the agarose gel showing a 75% level of purity (Fig. S5).

### 3.2 Optimization of 10,563 nt ssDNA yield in shake flask

**Fig. 3:**
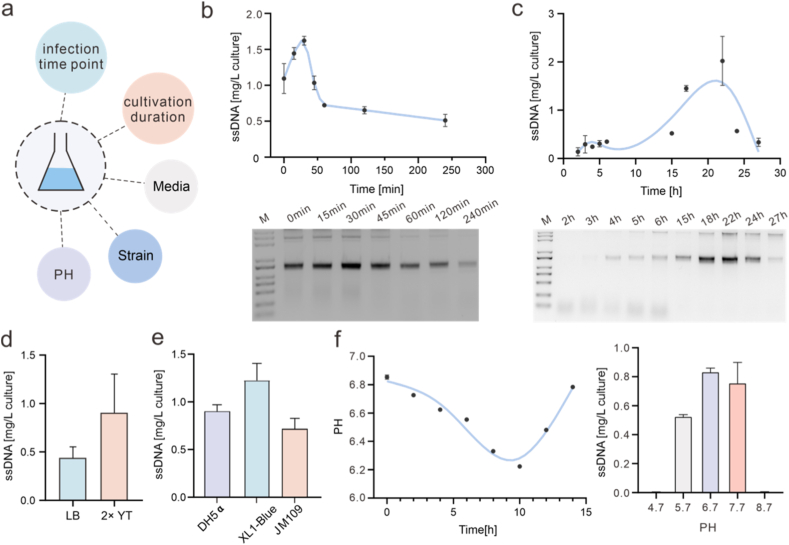
Systematic optimization of 10,563 nt ssDNA production in shake-flask. (a) Shaker flask growth was used to optimize conditions for phage amounts. (b) Time-course assay of ssDNA production showing that infection of *E. coli* with the helper phage at the 30 min timepoint after culture initiation resulted in the highest ssDNA yield. (c) Time-course assay of cssDNA production of 10,563 nt, with cssDNA yield calculated by absorbance at nanodrop (280 nm) and purity adjusted by agarose gel band intensity, showing maximum yield and purity at the 22 h timepoint. (d) Comparison between growth media showing two-fold improved cssDNA yield in 2 × YT after 22 h of production. (e) Comparison of ssDNA yield among DH5α, XL1-Blue, and JM109 strains. (f) Variation of culture pH under uncontrolled conditions (left). Comparison of ssDNA yield at pH 4.7, 5.7, 6.7, 7.7, and 8.7 (right). All data are presented as mean ± SD (n = 3 biological replicates). Error bars represent standard deviations.

To achieve high production of 10,563 nt ssDNA under shake flask conditions, we systematically evaluated five key factors: the timing of helper phage infection, cultivation duration, medium type, *E. coli* strain, and medium pH (Fig. 3a).

In helper phage-mediated phagemid ssDNA production systems, the multiplicity of infection (MOI) is one of the critical factors determining the efficiency of helper phage infection and subsequent ssDNA replication and packaging [24]. To determine the optimal infection time point, we investigated the effect of M13KO7 helper phage infection time on ssDNA yield. NanoDrop quantification results showed that ssDNA yield increased with infection time initially, then decreased, reaching its maximum at 30 min (Fig. 3b, upper panel). Agarose gel electrophoresis analysis (Fig. 3b, lower panel) further confirmed that 30 min was the optimal infection time point. Considering that extended cultivation time may affect both phage replication fidelity and particle recovery efficiency [25], we further examined the effect of total cultivation time on ssDNA yield. NanoDrop quantification results showed that ssDNA yield increased with cultivation time initially and then decreased, with a peak at 22 h (Fig. 3c, upper panel). Agarose gel electrophoresis analysis showed a similar trend, further confirming that 22 h of cultivation yielded the highest ssDNA output (Fig. 3c, lower panel).

Moreover, given that nutrient conditions significantly affect phage production, we assessed the impact of medium composition on ssDNA yield. In the phage-assisted rolling-circle replication ssDNA biosynthesis system, medium components, especially the concentrations of tryptone and yeast extract, play a critical role in determining ssDNA yield, with nutrient-rich media generally enhancing production efficiency [26]. Accordingly, we compared the effects of the nutrient-rich 2 × YT medium with standard LB medium. Both agarose gel electrophoresis and NanoDrop quantification results showed that the 2 × YT medium enabled the recovery of significantly higher amounts of extractable ssDNA compared to the LB medium (Fig. 3d and S6).

In addition to culture conditions, the choice of host strain also significantly influences phage infection efficiency, the stability of rolling-circle replication, and phage particle assembly and secretion [27]. Therefore, we compared three *E. coli* strains commonly used for ssDNA biosynthesis: DH5α, XL1-Blue, and JM109. NanoDrop quantification and agarose gel electrophoresis results were consistent (Fig. 3e and S7), indicating that XL1-Blue produced the highest ssDNA yield among the tested strains.

Finally, we investigated the impact of culture pH on filamentous phage production. Monitoring the pH dynamics during cultivation revealed a gradual decrease from an initial value of 6.87 to 6.22, followed by partial recovery (Fig. 3f, left panel). Culture pH has been shown to significantly affect phagemid production [28,29], and near-neutral pH conditions are favorable for bacterial growth and efficient filamentous phage replication and assembly [30]. Subsequently, the culture pH was controlled at 4.7, 5.7, 6.7, 7.7, and 8.7 to further evaluate its effect on ssDNA production. The ssDNA yield at each pH condition was quantified using NanoDrop measurements and further verified by agarose gel electrophoresis (Fig. 3f, right panel and Fig. S8). Among the tested conditions, pH 6.7 consistently produced the highest ssDNA yield.

### 3.3 The biosynthesis of 10,563 nt ssDNA in bioreactor

**Fig. 4:**
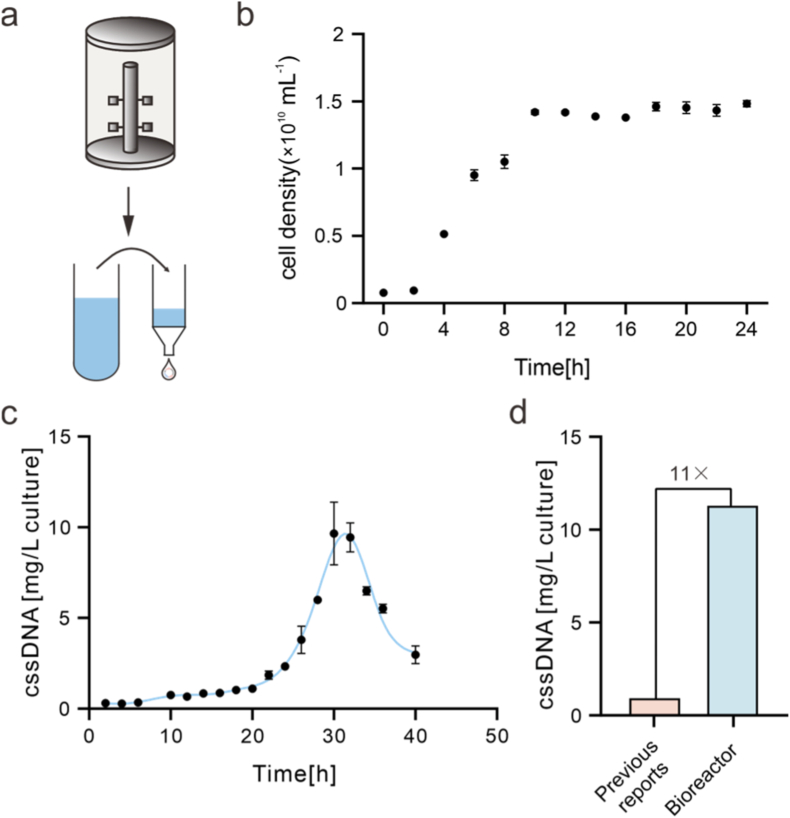
Bioreactor production of 10,563 nt ssDNA. (a) Schematic illustration of ssDNA biosynthesis in a bioreactor system. (b) Growth curve of *E. coli* during bioreactor cultivation, showing cell density as a function of cultivation time. (c) Effect of bioreactor cultivation time on ssDNA yield, showing a maximum ssDNA production at 30 h of cultivation. All data are presented as mean ± SD (n = 3 biological replicates). Error bars represent standard deviations. (d) Comparison of ssDNA yields obtained using the bioreactor-based approach and reported methods [22].

After systematically determining the optimal growth conditions in shake-flask cultures, we applied these conditions to a bioreactor system to achieve more precise and reproducible control over the key parameters governing ssDNA biosynthesis, enabling large-scale production (Fig. 4a). In contrast to shake-flask cultivation, the bioreactor allows real-time monitoring and precise regulation of dissolved oxygen (DO) levels. Both insufficient and excessive DO levels can adversely affect phage-related processes, highlighting the necessity of strict DO control to ensure efficient ssDNA production [31].

We first characterized the growth behavior of *E. coli* in the bioreactor (Fig. 4b). Growth kinetics revealed that the culture entered a stable growth phase approximately 10 h after inoculation, providing an appropriate window for sustained phagemid replication and ssDNA production. Based on this growth profile, we further investigated the effect of cultivation time in the bioreactor on ssDNA yield. NanoDrop quantification showed that ssDNA production gradually increased with cultivation time, reached a maximum at 30 h, and subsequently declined (Fig. 4c). Under bioreactor controlled conditions, the yield of biosynthesized ssDNA reached 11.35 mg L^−1^. Notably, this yield represents an approximately tenfold increase compared with the ∼1 mg L^−1^ yield previously reported for ssDNA of similar length (Fig. 4d) [20], highlighting the effectiveness of bioreactor-based process control for enhancing long ssDNA production.

### 3.4 The construction of large DNA origami nanostructures

We constructed large DNA origami nanostructures using biosynthesized 10,563 nt ssDNA as the scaffold. Based on the square-lattice design principle implemented in the caDNAno software [32], we designed a large triangular DNA origami nanostructure (the edge length of 147.8 nm [32]) composed of multiple trapezoidal domains with a crossover spacing of 32 base pairs. The slanted edges of adjacent trapezoidal domains converge at the triangle vertices, enabling the incorporation of bridging staple strands at these interfaces, which effectively stabilizes the vertex regions and yields discrete triangular structures with uniform geometry and consistent angles. Using the 10,563 nt scaffold also increases the number of available staple binding positions compared with the conventional M13mp18 scaffold (7249 nt). Based on the staple routing in our design, this corresponds to approximately 100 additional potential functionalization sites that could be introduced through staple modification [33,34]. In addition, we constructed a large rectangular DNA origami structure (86.3 × 138.5 nm, the inter-helix gap of 1.5 nm [32]) following the design reported by Chen et al. which features a crossover spacing of 128 base pairs [20]. However, the effects of the staple-to-scaffold ratio and scaffold concentration on the folding efficiency of such large-scale DNA origami structures have not yet been systematically investigated. Detailed design parameters and schematic illustrations are provided in Figs. S9 and S10.

**Fig. 5:**
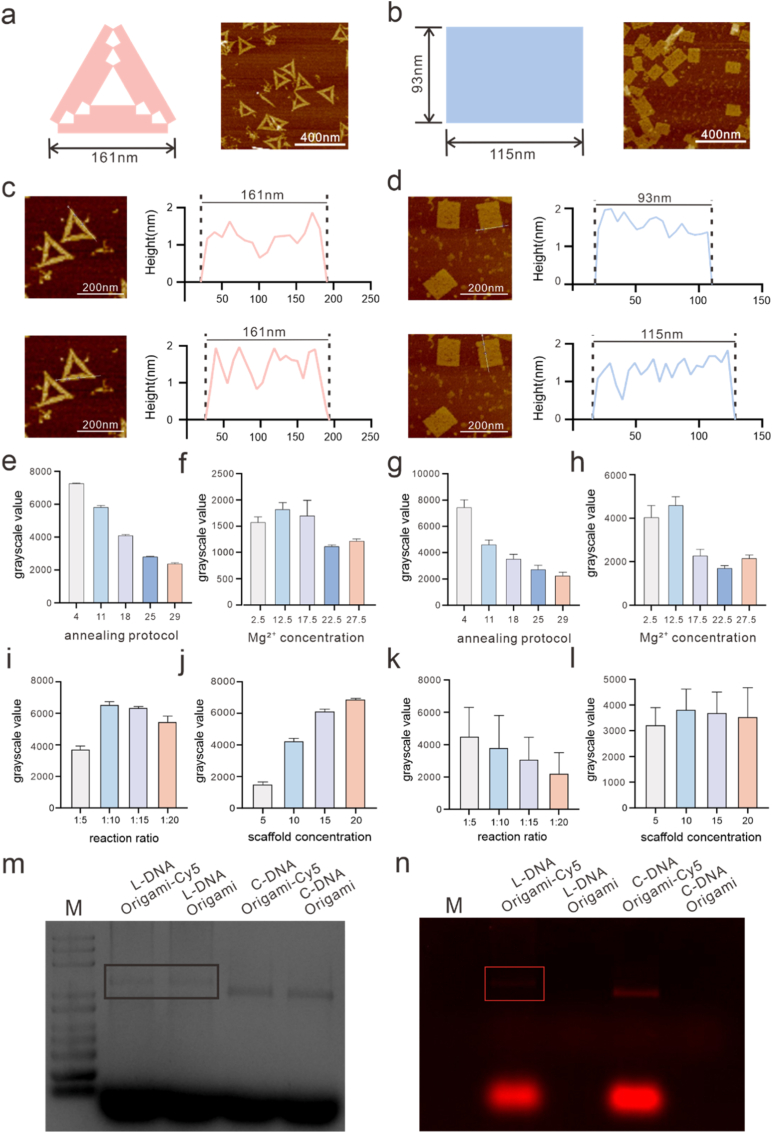
Construction of large-scale DNA origami nanostructures using 10,563 nt ssDNA. (a) AFM characterization of large triangular DNA origami structures constructed using 10,563 nt ssDNA as the scaffold strand. (b) AFM characterization of large rectangular DNA origami structures constructed using 10,563 nt ssDNA as the scaffold strand. (c) AFM characterization of large triangular DNA origami structures constructed using 10,563 nt ssDNA as the scaffold strand, showing an equilateral triangular geometry with an edge length of 161 nm. (d) AFM characterization of large rectangular DNA origami structures constructed using 10,563 nt ssDNA as the scaffold strand, exhibiting a length of 115 nm and a width of 93 nm. (e-f) Effect of Mg^2+^ concentration and annealing protocol on the assembly of large triangular DNA origami. g-h) Effect of Mg^2+^ concentration and annealing protocol on the assembly of large rectangular DNA origami. (i-j) Quantitative ImageJ analysis of agarose gel electrophoresis bands indicating that the large triangular DNA origami structures exhibited maximal yield at a reaction stoichiometry of 1:10 with a scaffold concentration of 20 nM. (k-l) Quantitative ImageJ analysis of agarose gel electrophoresis bands showing that the large rectangular DNA origami structures achieved the highest yield at a reaction stoichiometry of 1:5 with a scaffold concentration of 10 nM. (m) Agarose gel electrophoresis of DNA origami visualized under UV illumination. (n) Agarose gel electrophoresis of DNA origami visualized under Cy5 fluorescence excitation.

Large DNA origami nanostructures were successfully fabricated using a programmed thermal annealing protocol. Atomic force microscopy (AFM) characterization demonstrated that both large triangular and rectangular DNA origami structures were robustly assembled (Fig. 5a and b; uncropped images are shown in Figs. S11 and S12), exhibiting high structural integrity and good morphological uniformity. Further statistical analysis revealed that the triangular DNA origami had an average edge length of 161 ± 11 nm (mean ± SD, N = 80) (Fig. 5c and S13), with approximately 72% of the analyzed structures classified as well-formed. This dimension is larger than that of triangular DNA origami assembled using the conventional M13mp18 scaffold, which typically exhibits an edge length of approximately 120 nm. In addition, the rectangular DNA origami assembled using the biosynthesized scaffold exhibited average dimensions of 115 ± 7 nm in length and 93 ± 5 nm in width (mean ± SD, N = 70) (Fig. 5d and S14). Approximately 75% of the analyzed structures were well-formed, indicating good structural integrity. These dimensions are also larger than those typically obtained using the M13mp18 scaffold (≈60 × 90 nm). It should be noted that unavoidable drift effects during AFM imaging can distort the aspect ratio of the structures, resulting in deviations between the experimentally measured dimensions and the designed values [33,35]. Meanwhile, we observed that large DNA origami assembled using the biosynthesized 10,563 nt scaffold exhibited a lower overall folding efficiency compared to DNA origami folded from the conventional M13mp18 scaffold. This reduced folding efficiency is likely attributable to the increased propensity of longer scaffold strands to form intramolecular secondary structures, which can adversely affect folding kinetics and ultimately decrease folding efficiency and effective yield [23,36]. Secondary structure predictions were performed for both the conventional M13mp18 scaffold and the 10,563 nt biosynthesized scaffold (Figs. S15 and S16). The longer scaffold exhibited a more complex branched secondary structure and a more negative predicted folding free energy, indicating a higher propensity for intramolecular base pairing. These features suggest that the longer scaffold may present additional structural constraints during DNA origami assembly.

To improve the folding efficiency of the large DNA origami structures, the effects of Mg^2+^ concentration and annealing protocol on DNA origami assembly were first investigated. Agarose gel electrophoresis analysis using ImageJ (Fig. 5e–h and S17-S18) showed that the strongest origami band was obtained at 12.5 mM Mg^2+^, indicating that folding of the long scaffold is highly sensitive to ionic conditions. In addition, increasing the annealing time reduced dimer formation in the triangular origami, although the intensity of the monomer band gradually decreased. For the rectangular origami, prolonged annealing also resulted in weaker origami bands. Based on these results, a Mg^2+^ concentration of 12.5 mM and an annealing time of 18 h were selected for the assembly of both triangular and rectangular DNA origami structures.

Motivated by previous studies indicating that folding efficiency is strongly influenced by the relative concentrations of scaffold and staple strands as well as the scaffold strand concentration itself [37,38], we further examined the effects of reaction stoichiometry and scaffold strand concentration on the assembly process. Folding yields of large DNA origami were evaluated by quantitative analysis of agarose gel electrophoresis bands using ImageJ (Figs. S19 and S20). For the large triangular DNA origami structures, the highest folding yield was achieved at a scaffold concentration of 20 nM with a staple-to-scaffold ratio of 10:1 (Fig. 5i and g). In contrast, the large rectangular DNA origami exhibited maximal yield at a staple-to-scaffold ratio of 5:1 and a scaffold concentration of 10 nM (Fig. 5k and l). These results demonstrate that the optimal conditions required for efficient folding differ markedly among DNA origami structures with distinct geometries. DNA origami assembly is governed by cooperative hybridization and design-dependent nucleation barriers, and architectures with higher structural complexity generally require greater staple excess and higher scaffold concentrations [39]. Because triangular DNA origami impose more stringent geometric constraints at their vertices than rectangular structures, they exhibit higher overall structural complexity and therefore require increased scaffold concentrations and higher staple-to-scaffold ratios to achieve efficient folding [32,40]. Collectively, our results quantitatively validate this concept and provide optimized experimental parameters for the preparation of large-scale triangular and rectangular DNA origami structures. In addition to the expected product bands, additional signals were observed near the top of the agarose gel lanes (Figs. S17–S20). These high-molecular-weight bands may correspond to multimeric DNA origami structures [33]. Consistently, multimeric assemblies were also observed in the AFM images. The formation of these multimers is likely related to the omission of staple strands at the vertical edges and vertices of the DNA origami, which leaves blunt-ended helices exposed and facilitates inter-structure stacking [32,35].

The large triangular DNA origami assembled using the 10,563 nt scaffold contains approximately 100 additional potential functionalization sites compared with the conventional triangular origami assembled using the M13mp18 scaffold. To verify the accessibility of these sites, Cy5-labeled staple strands were introduced at peripheral positions near the vertices of each edge of the triangular structures. For comparison, the same Cy5 modifications were introduced at the corresponding positions of the conventional triangular DNA origami (Fig. S21). The sequences of the Cy5-modified staples are listed in Table S5. The assembled DNA origami structures with and without Cy5 labels were analyzed by agarose gel electrophoresis (Fig. 5m and n). Under UV illumination, both structures showed clear bands corresponding to folded DNA origami. Under Cy5 excitation, fluorescence signals were observed only for the Cy5-labeled structures, confirming successful incorporation of the fluorescent staples. These results indicate that the additional peripheral sites in the large triangular DNA origami are accessible for functional modification.

## 4 Discussion

In summary, this study enhanced long ssDNA yield from a phage-mediated biosynthesis system by systematically optimizing biosynthesis conditions, achieving an approximately tenfold increase compared with previously reported methods. Subsequently, the biosynthesized ssDNA was employed as the scaffold strand for DNA origami assembly, based on which large triangular DNA origami structures were designed. Using this scaffold, both large triangular and large rectangular DNA origami nanostructures were successfully constructed, thereby demonstrating the general applicability and feasibility of the biosynthesized ssDNA as a robust scaffold source for building large DNA origami systems; notably, the resulting large-sized designs provide up to 100 additional potential functionalization sites. These results demonstrate that the quality and quantity of the biosynthesized ssDNA are sufficient to support the reliable construction of large DNA origami architectures.

Importantly, the major cost of this biosynthetic strategy is largely limited to the one-time construction of the recombinant phagemid. Once established, subsequent ssDNA production relies primarily on bacterial cultivation media and helper phage, making the overall process scalable and potentially cost-effective. Moreover, the future implementation of fed-batch cultivation strategies is expected to further improve ssDNA productivity. Collectively, these results underscore the advantages of the established ssDNA biosynthesis platform in terms of production efficiency, cost-effectiveness, and scalability, while providing a robust and sustainable scaffold source for the construction of DNA origami with larger dimensions and increased structural complexity. As such, this work lays a solid foundation for expanding the practical applications of DNA origami in nanofabrication, biosensing, and biomedicine.

## CRediT authorship contribution statement

**Meiling Lu:** Conceptualization, Data curation, Writing – original draft. **Xiwei Wang:** Conceptualization, Data curation, Writing – original draft. **Baohong He:** Conceptualization, Supervision, Validation. **Jingyan Zhang:** Conceptualization, Software, Supervision. **Youqing Cu:** Conceptualization, Supervision, Validation. **Jinjing Che:** Formal analysis. **Nan Liu:** Resources, Software, Supervision, Validation. **Zengming Wang:** Resources, Software, Supervision, Validation. **Hui Zhang:** Resources, Software, Supervision, Validation. **Liang Xu:** Resources, Software, Supervision, Validation. **Xuili Gao:** Funding acquisition, Resources, Supervision, Validation. **Aiping Zheng:** Funding acquisition, Resources, Supervision, Validation.

## Declaration of competing interest

The authors declare that they have no known competing financial interests or personal relationships that could have appeared to influence the work reported in this paper.

## Data Availability

No data was used for the research described in the article.
