# Supplementary material for: Bioproduction of ∼10 knt single-stranded DNA for constructing large DNA origami structures
Source: Mater Today Bio. 2026 Apr 14;38:103092. doi: 10.1016/j.mtbio.2026.103092 (PMC13147996; doi:10.1016/j.mtbio.2026.103092)
Supplement: Multimedia component 1 [file mmc1.docx]

**Bioproduction of Hundred Thousand Bases Single-Stranded DNA for Constructing Large DNA Origami Structures**

Meiling Lu^a,b,#^, Xiwei Wang^b,#^, Baohong He^b^, Jingyan Zhang^b^, Youqing Cu^b^, Jinjing Che^b^, Nan Liu^b^, Zengming Wang^b^, Hui Zhang^b^, Liang Xu^b^, Xuili Gao^a,^*, Aiping Zheng^b,^*

*^a^ State Key Laboratory of Discovery and Utilization of Functional Components in Traditional Chinese Medicine, Guizhou Medical University, Guiyang 550000, China*

*^b^ Academy of Military Medical Sciences, Beijing 100850, China*

^#^These authors contributed equally to this work

*Corresponding author

E-mail addresses: [apzheng@163.com](mailto:apzheng@163.com), gaoxl@gmc.edu.cn


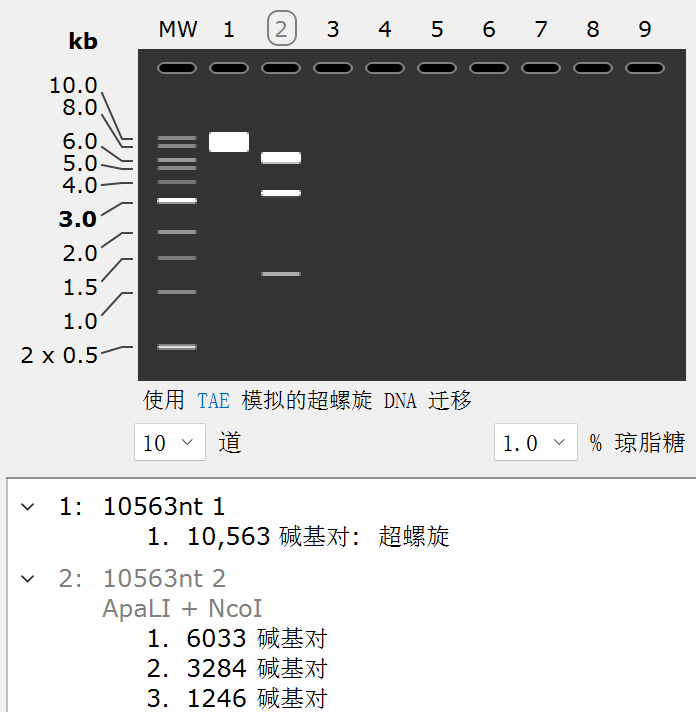


Figure S1. Restriction digestion of the 10,563 bp recombinant phagemid with ApaLI and NcoI was simulated using SnapGene, predicting the generation of three fragments of 1,246 bp, 3,284 bp, and 6,033 bp.

**
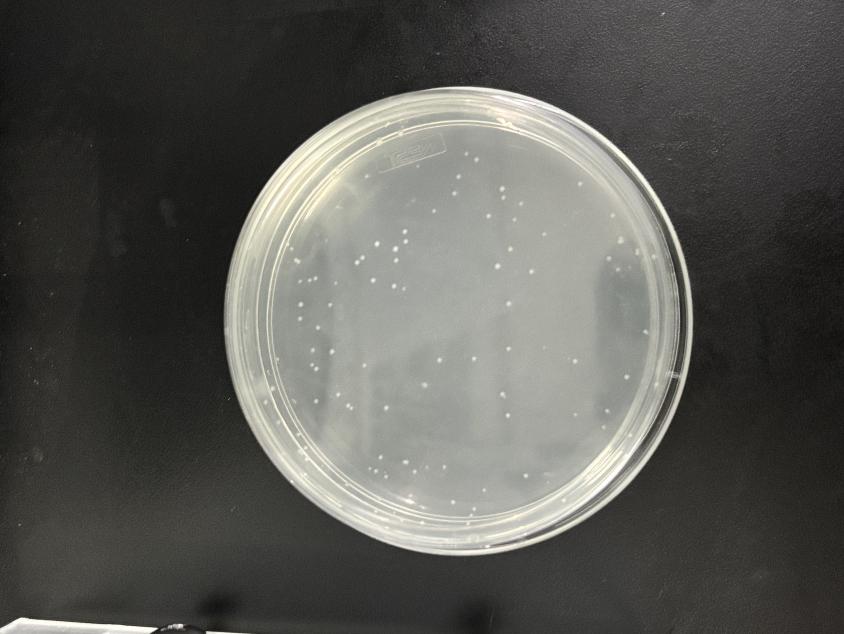
**

Figure S2. Colony formation of E. coli harboring the recombinant phagemid on ampicillin-containing agar plates.


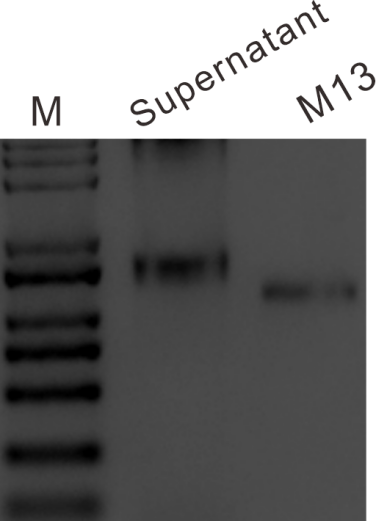


Figure S3. Agarose gel electrophoresis results showed that the left band corresponded to ssDNA with a length of 10,563 nt, while the right band represented ssDNA derived from M13mp18 with a length of 7,249 nt.


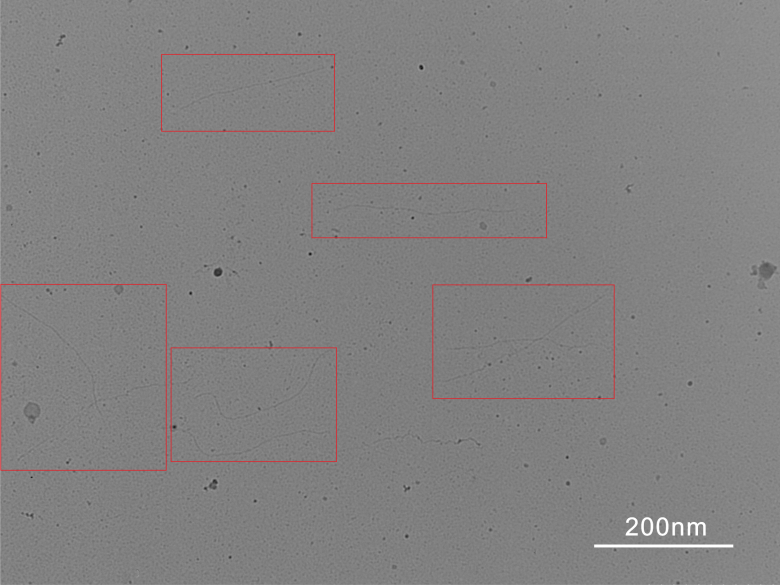


Figure S4. Wide-field TEM micrograph showing assembled phage in the clarified media, rod-shaped and approximately 200 nm in size.


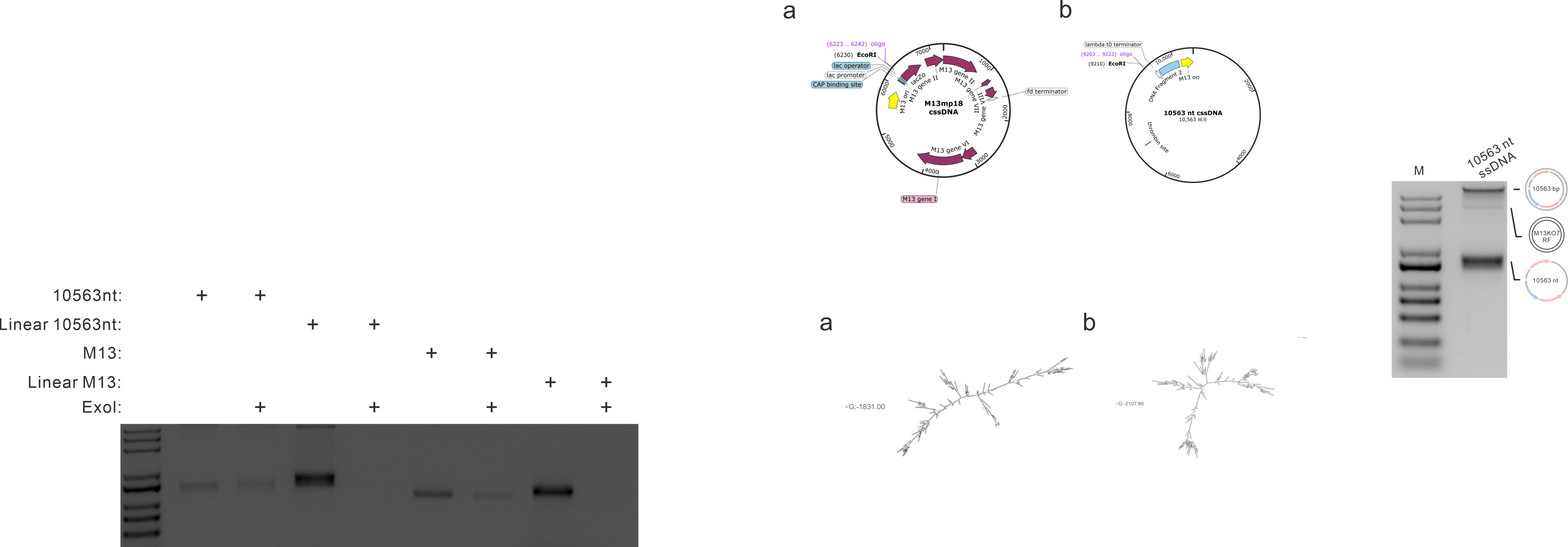


Figure S5. Agarose gel electrophoresis image used for purity analysis of the biosynthesized 10,563 nt ssDNA.


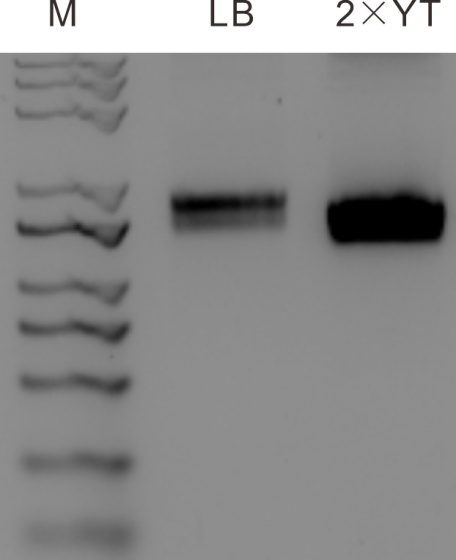


Figure S6. The effect of LB and 2×YT media on ssDNA yield was characterized by agarose gel electrophoresis.


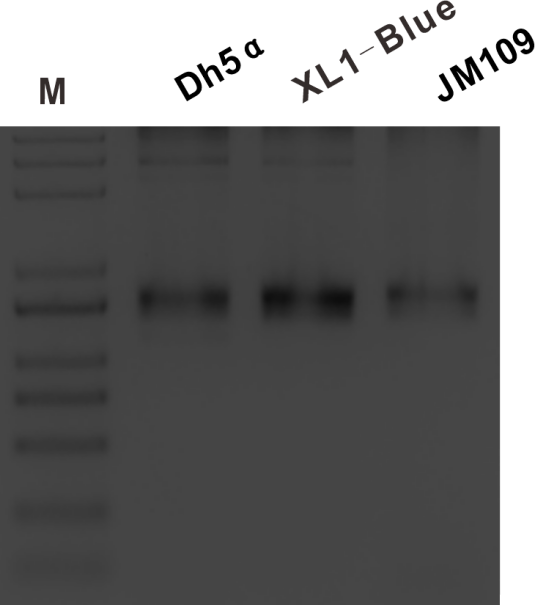


Figure S7. The effect of strain on cssDNA yield was characterized by agarose gel electrophoresis.


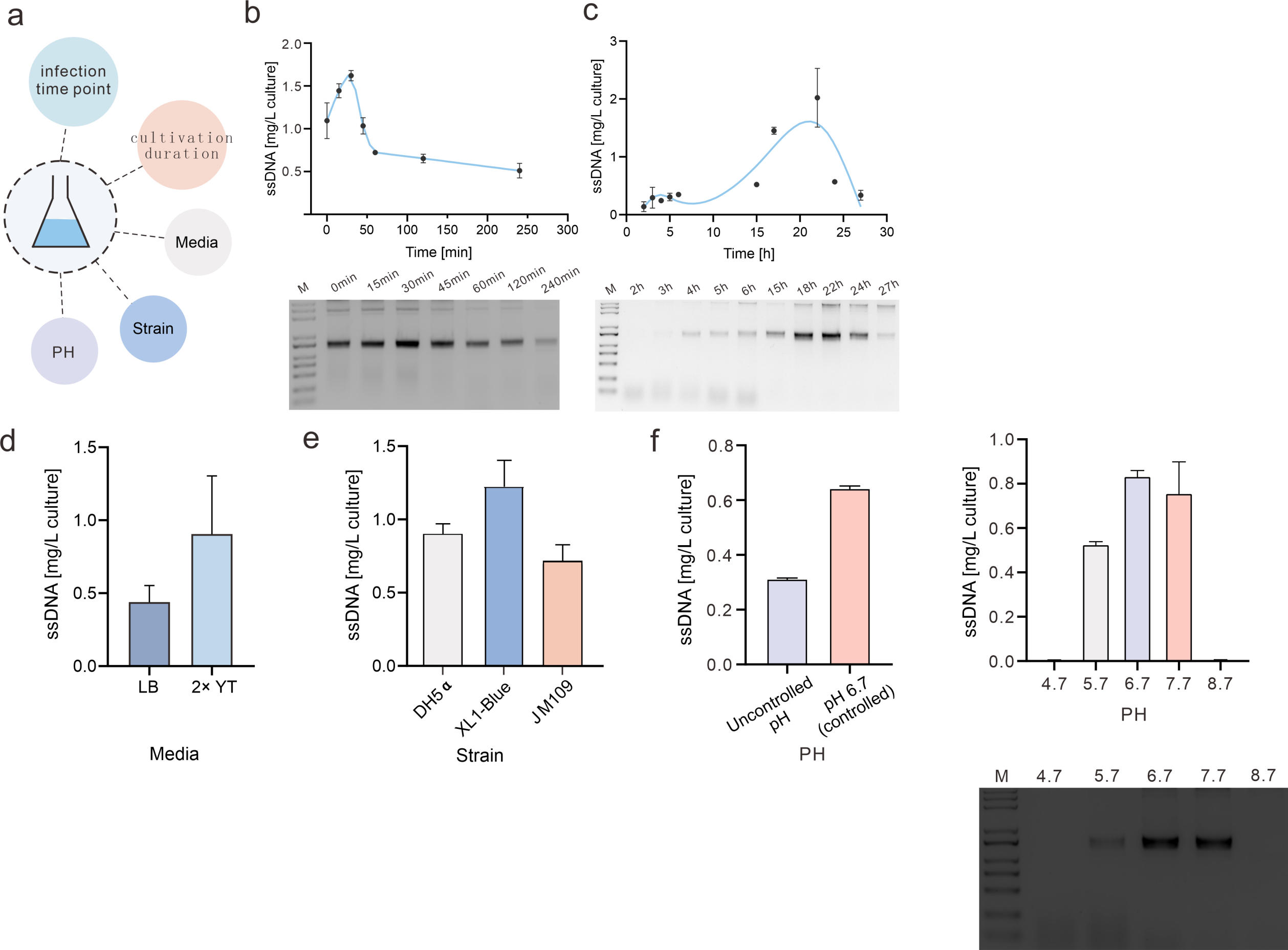


Figure S8. The effect of PH on cssDNA yield was characterized by agarose gel electrophoresis.


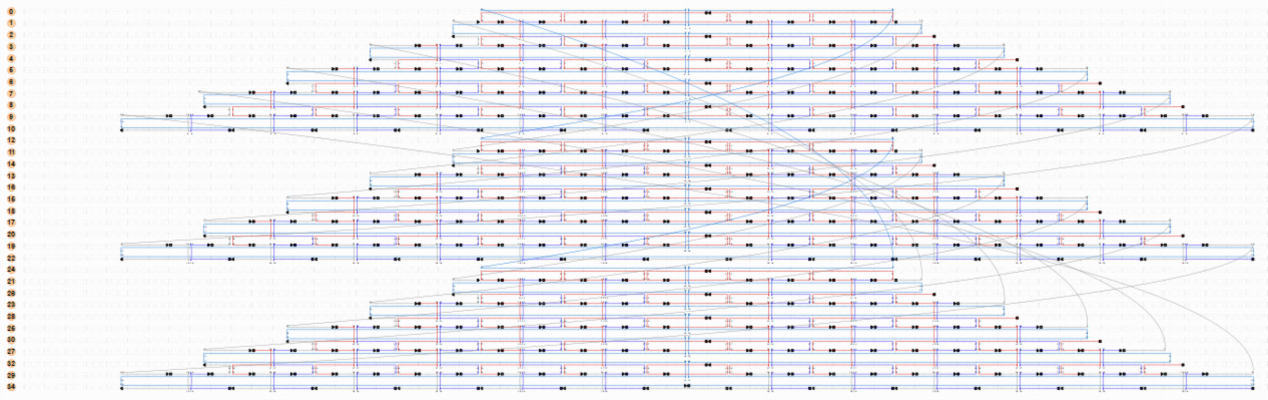


Figure S9. The strand diagram of large triangular DNA origami structure designed based on a 10,563 nt scaffold.


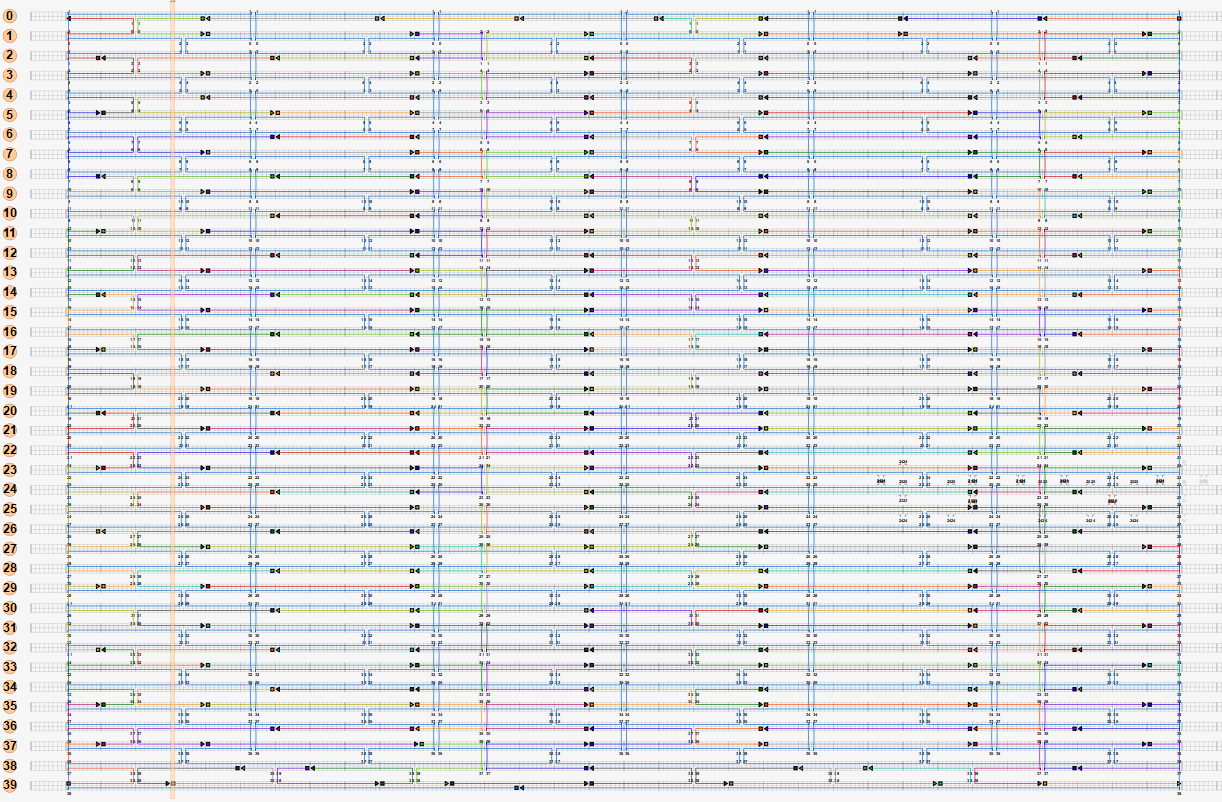


Figure S10. The strand diagram of large rectangular DNA origami structure designed based on a 10,563 nt scaffold.


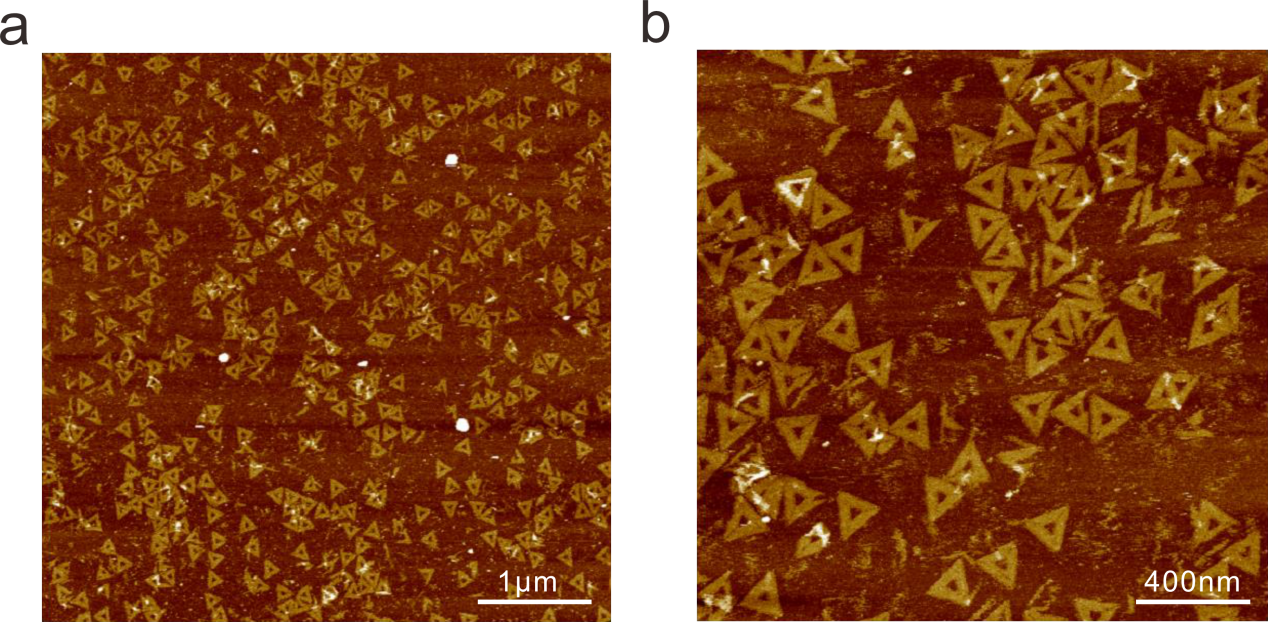


Figure S11. AFM images of the large triangular DNA origami folded from 10563 nt.

Scale bar: 1μm and 400 nm.


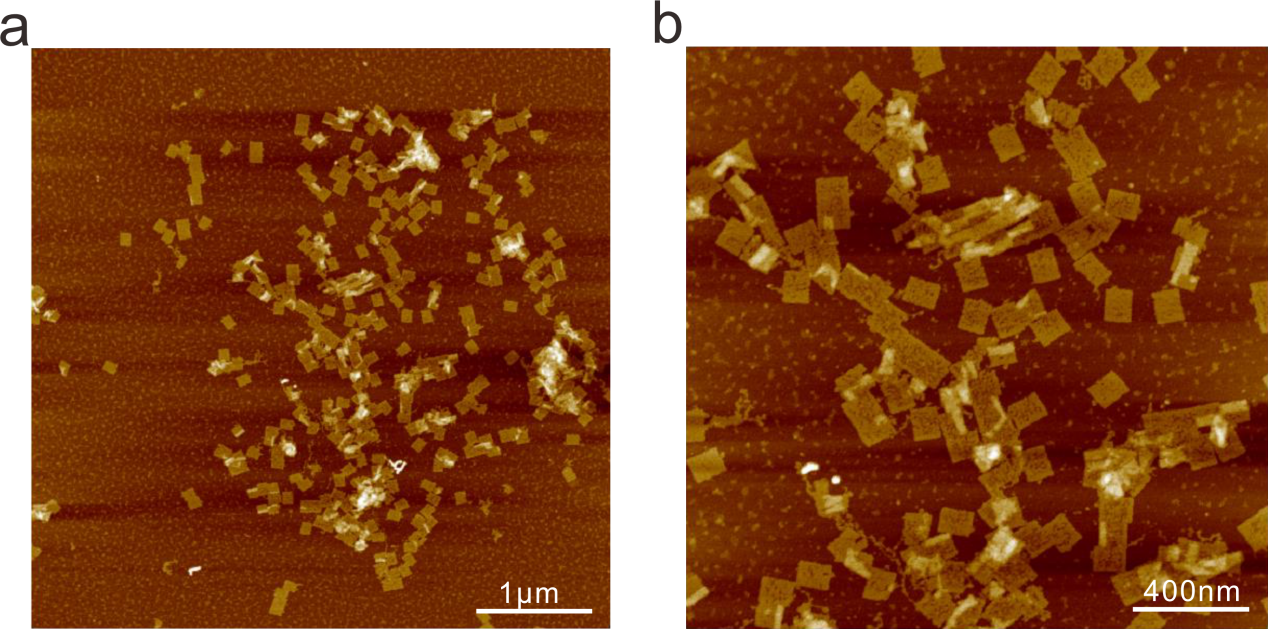


Figure S12. AFM images of the large rectangular DNA origami folded from 10563 nt.

Scale bar: 1μm and 400 nm.


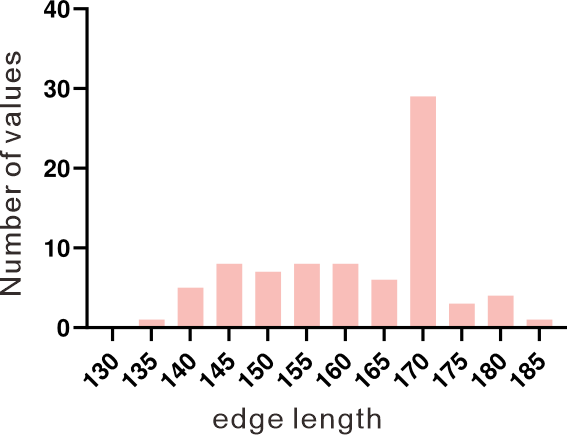


Figure S13. Histogram of edge length distribution for the large triangular DNA origami.


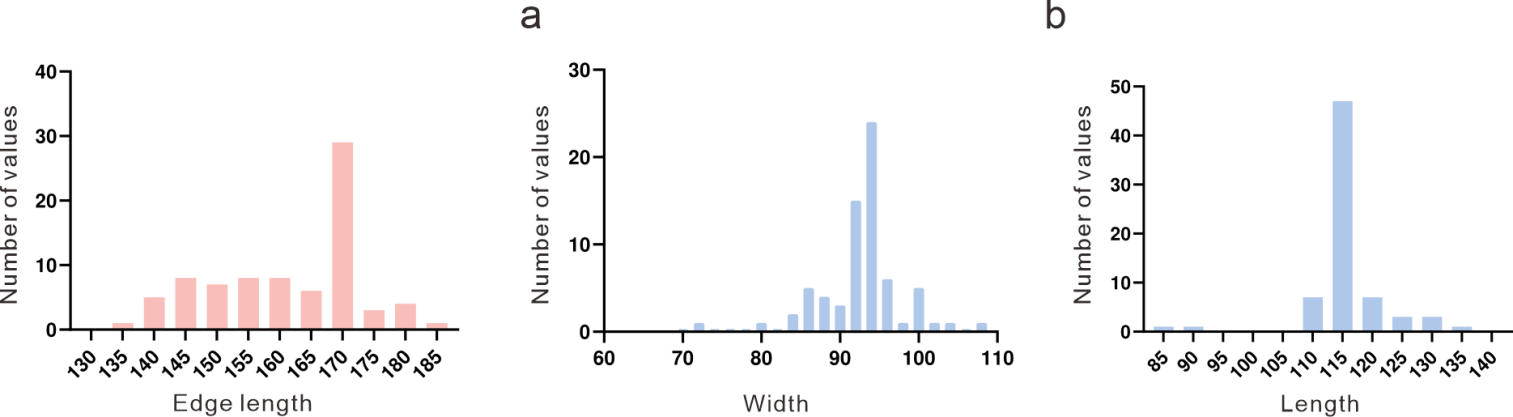


Figure S14. Histogram of width and length distribution for the large rectangular DNA origami.


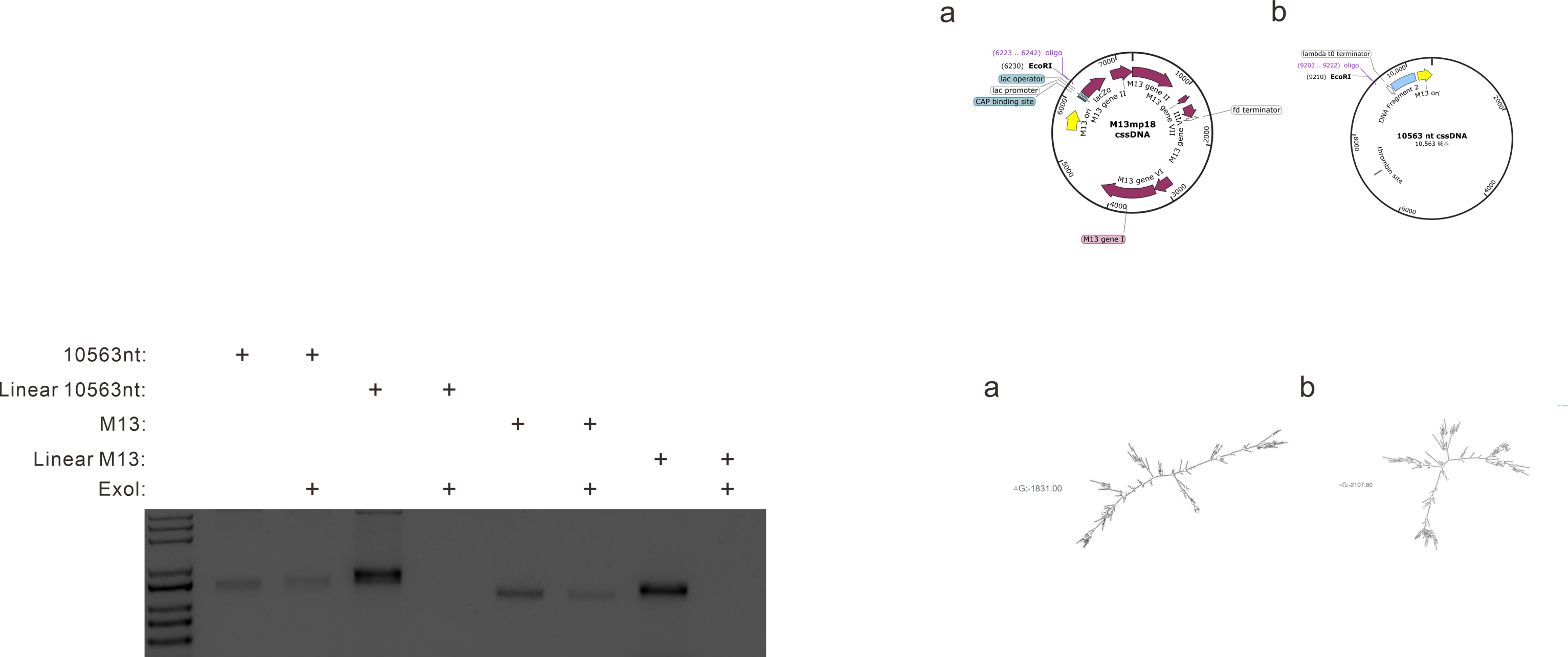


Figure S15. secondary structure prediction of the 10,563 nt scaffold.


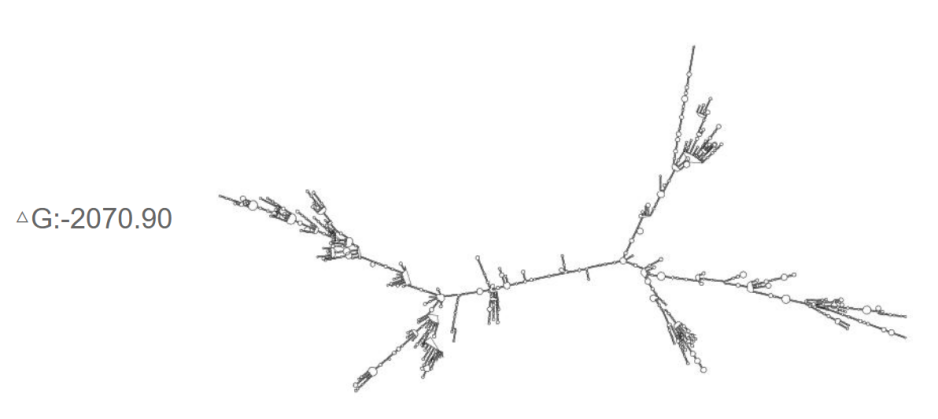


Figure S16. secondary structure prediction of the M13mp18 scaffold.


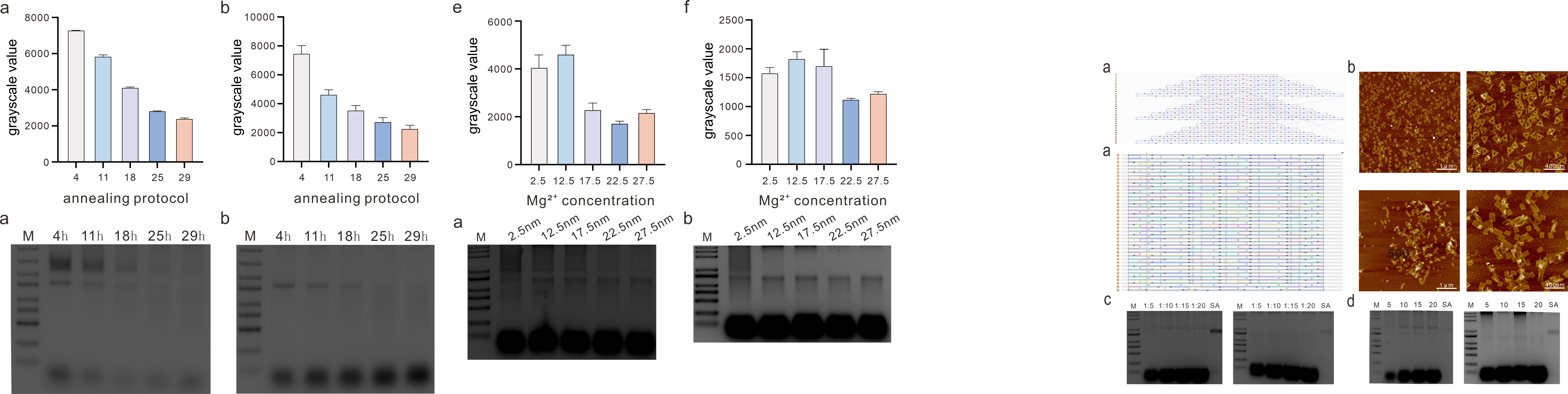


Figure S17. (a) Agarose gel electrophoresis analysis showing the effect of annealing protocol on the yield of large triangular DNA origami. (b) Agarose gel electrophoresis analysis showing the effect of annealing protocol on the yield of large rectangular DNA origami.


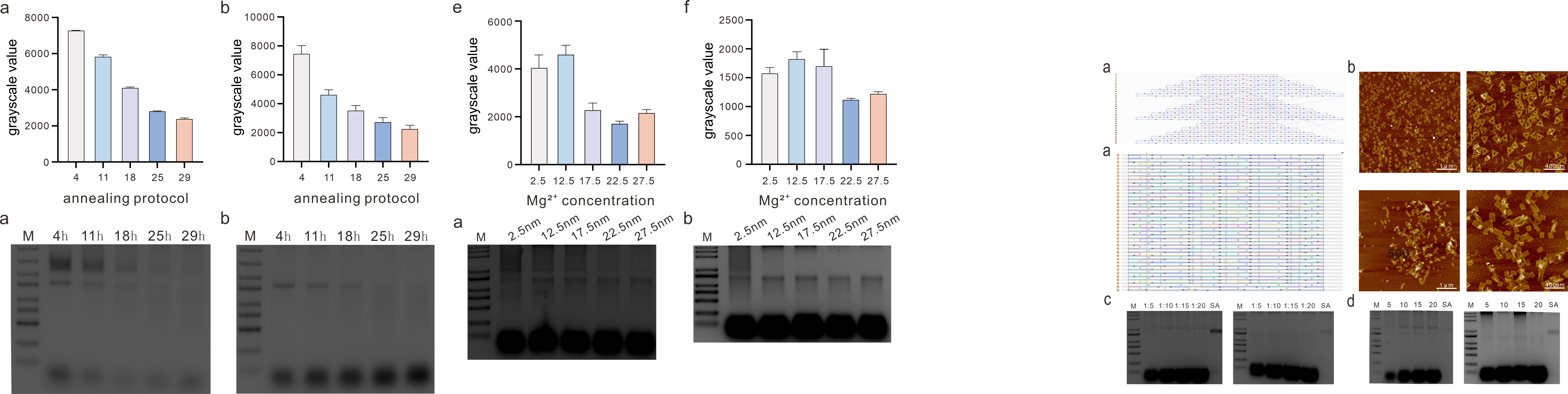


Figure S18. (a) Agarose gel electrophoresis analysis showing the effect of Mg²⁺ concentration on the yield of large triangular DNA origami. (b) Agarose gel electrophoresis analysis showing the effect of Mg²⁺ concentration on the yield of large rectangular DNA origami.


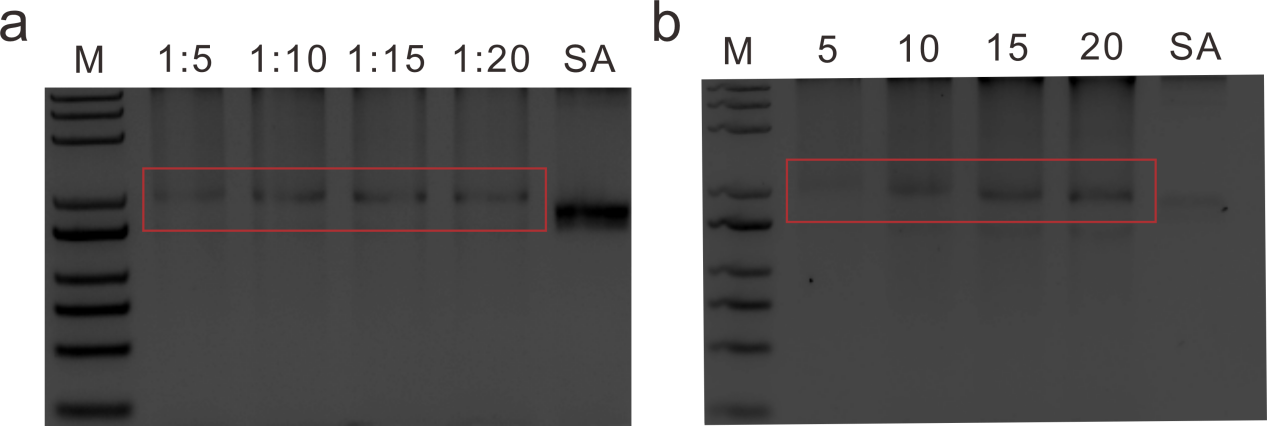


Figure S19. (a) Agarose gel electrophoresis analysis of large triangular DNA origami assembled under different reaction stoichiometries. (b) Agarose gel electrophoresis analysis of large rectangular DNA origami assembled at different scaffold strand concentrations.


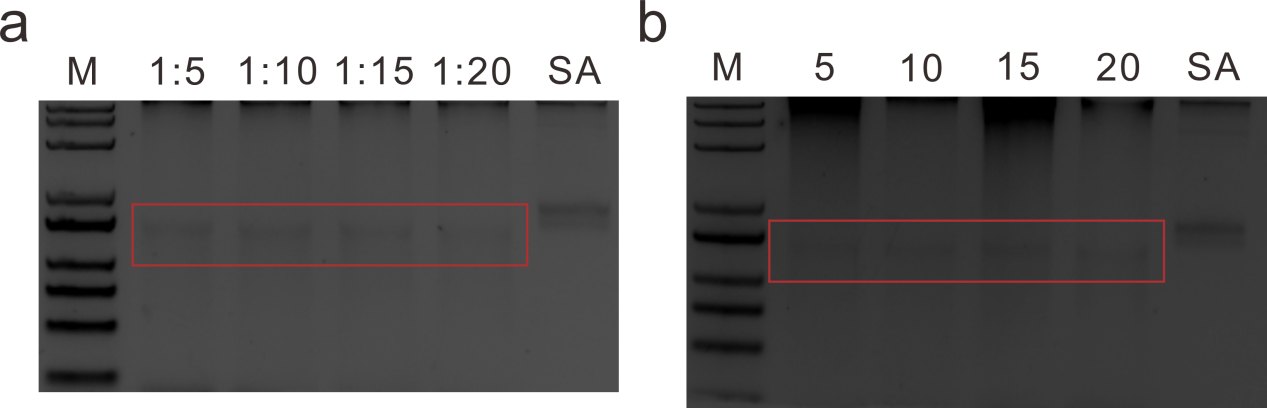


Figure S20. (a) Agarose gel electrophoresis analysis of large rectangular DNA origami assembled under different reaction stoichiometries. (b) Agarose gel electrophoresis analysis of large rectangular DNA origami assembled at different scaffold strand concentrations.

**
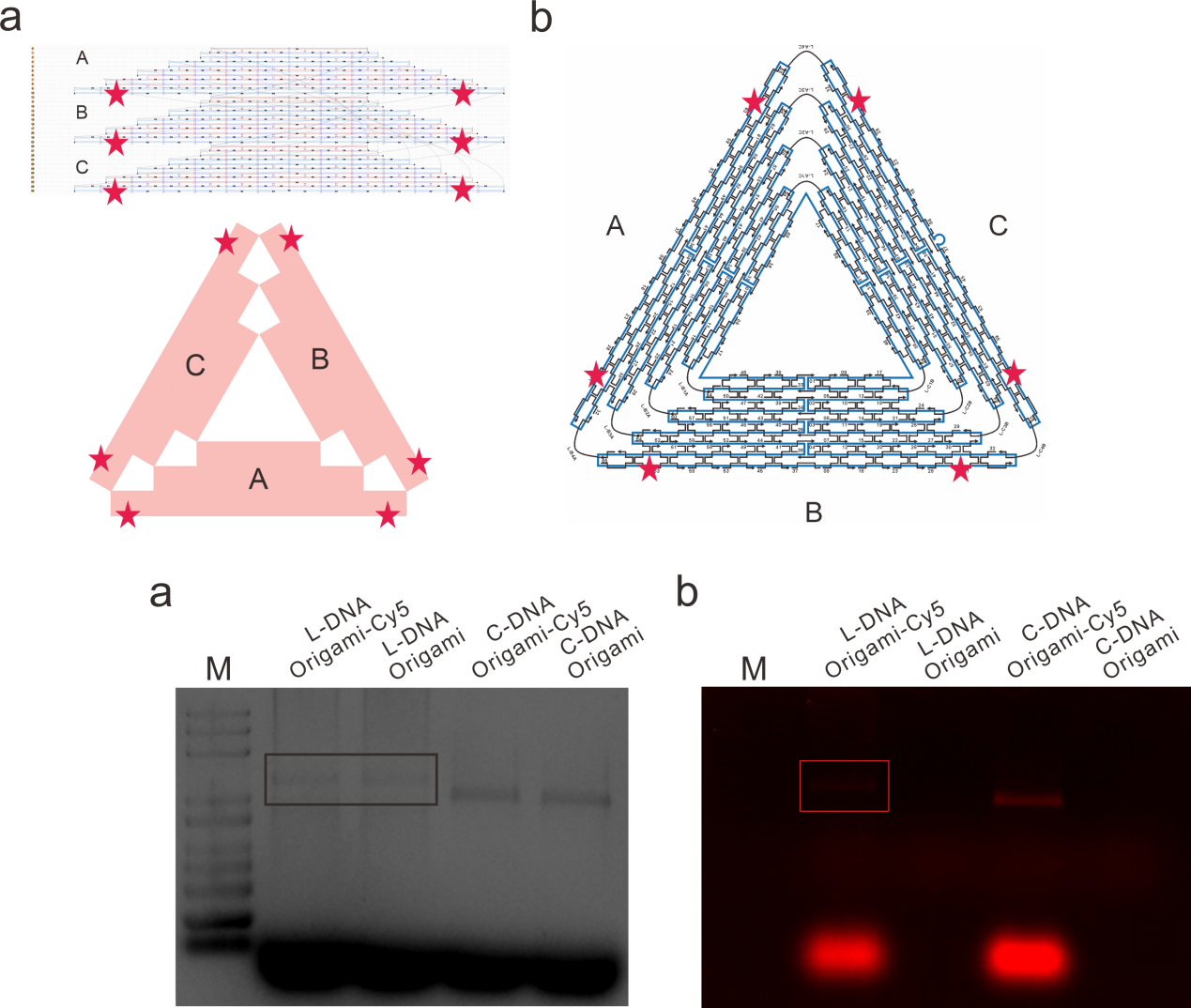
**

Figure S21. Schematic illustration of Cy5 labeling on the DNA origami structure.

Table S1. Sequence of the 10,563 nt ssDNA

| 10,563 nt ssDNA sequence: |
| --- |
| TTTTGCCGATTTCGGGTTTTCGCTATTTATGAAAATTTTCCGGTTTAAGGCGTTTCCGTTCTTCTTCGTCATAACTTAATGTTTTTATTTAAAATACCCTCTGAAAAGAAAGGAAACGACAGGTGCTGAAAGCGAGGCTTTTTGGCCTCTGTCGTTTCCTTTCTCTGTTTTTGTCCGTGGAATGAACAATGGAAGTCAACAAAAAGCAGCTGGCTGACATTTTCGGTGCGAGTATCCGTACCATTCAGAACTGGCAGGAACAGGGAATGCCCGTTCTGCGAGGCGGTGGCAAGGGTAATGAGGTGCTTTATGACTCTGCCGCCGTCATAAAATGGTATGCCGAAAGGGATGCTGAAATTGAGAACGAAAAGCTGCGCCGGGAGGTTGAAGAACTGCGGCAGGCCAGCGAGGCAGATCTCCAGCCAGGAACTATTGAGTACGAACGCCATCGACTTACGCGTGCGCAGGCCGACGCACAGGAACTGAAGAATGCCAGAGACTCCGCTGAAGTGGTGGAAACCGCATTCTGTACTTTCGTGCTGTCGCGGATCGCAGGTGAAATTGCCAGTATTCTCGACGGGCTCCCCCTGTCGGTGCAGCGGCGTTTTCCGGAACTGGAAAACCGACATGTTGATTTCCTGAAACGGGATATCATCAAAGCCATGAACAAAGCAGCCGCGCTGGATGAACTGATACCGGGGTTGCTGAGTGAATATATCGAACAGTCAGGTTAACAGGCTGCGGCATTTTGTCCGCGCCGGGCTTCGCTCACTGTTCAGGCCGGAGCCACAGACCGCCGTTGAATGGGCGGATGCTAATTACTATCTCCCGAAAGAATCCGCATACCAGGAAGGGCGCTGGGAAACACTGCCCTTTCAGCGGGCCATCATGAATGCGATGGGCAGCGACTACATCCGTGAGGTGAATGTGGTGAAGTCTGCCCGTGTCGGTTATTCCAAAATGCTGCTGGGTGTTTATGCCTACTTTATAGAGCATAAGCAGCGCAACACCCTTATCTGGTTGCCGACGGATGGTGATGCCGAGAACTTTATGAAAACCCACGTTGAGCCGACTATTCGTGATATTCCGTCGCTGCTGGCGCTGGCCCCGTGGTATGGCAAAAAGCACCGGGATAACACGCTCACCATGAAGCGTTTCACTAATGGGCGTGGCTTCTGGTGCCTGGGCGGTAAAGCGGCAAAAAACTACCGTGAAAAGTCGGTGGATGTGGCGGGTTATGATGAACTTGCTGCTTTTGATGATGATATTGAACAGGAAGGCTCTCCGACGTTCCTGGGTGACAAGCGTATTGAAGGCTCGGTCTGGCCAAAGTCCATCCGTGGCTCCACGCCAAAAGTGAGAGGCACCTGTCAGATTGAGCGTGCAGCCAGTGAATCCCCGCATTTTATGCGTTTTCATGTTGCCTGCCCGCATTGCGGGGAGGAGCAGTATCTTAAATTTGGCGACAAAGAGACGCCGTTTGGCCTCAAATGGACGCCGGATGACCCCTCCAGCGTGTTTTATCTCTGCGAGCATAATGCCTGCGTCATCCGCCAGCAGGAGCTGGACTTTACTGATGCCCGTTATATCTGCGAAAAGACCGGGATCTGGACCCGTGATGGCATTCTCTGGTTTTCGTCATCCGGTGAAGAGATTGAGCCACCTGACAGTGTGACCTTTCACATCTGGACAGCGTACAGCCCGTTCACCACCTGGGTGCAGATTGTCAAAGACTGGATGAAAACGAAAGGGGATACGGGAAAACGTAAAACCTTCGTAAACACCACGCTCGGTGAGACGTGGGAGGCGAAAATTGGCGAACGTCCGGATGCTGAAGTGATGGCAGAGCGGAAAGAGCATTATTCAGCGCCCGTTCCTGACCGTGTGGCTTACCTGACCGCCGGTATCGACTCCCAGCTGGACCGCTACGAAATGCGCGTATGGGGATGGGGGCCGGGTGAGGAAAGCTGGCTGATTGACCGGCAGATTATTATGGGCCGCCACGACGATGAACAGACGCTGCTGCGTGTGGATGAGGCCATCAATAAAACCTATACCCGCCGGAATGGTGCAGAAATGTCGATATCCCGTATCTGCTGGGATACTGGCGGGATTGACCCGACCATTGTGTATGAACGCTCGAAAAAACATGGGCTGTTCCGGGTGATCCCCATTAAAGGGGCATCCGTCTACGGAAAGCCGGTGGCCAGCATGCCACGTAAGCGAAACAAAAACGGGGTTTACCTTACCGAAATCGGTACGGATACCGCGAAAGAGCAGATTTATAACCGCTTCACACTGACGCCGGAAGGGGATGAACCGCTTCCCGGTGCCGTTCACTTCCCGAATAACCCGGATATTTTTGATCTGACCGAAGCGCAGCAGCTGACTGCTGAAGAGCAGGTCGAAAAATGGGTGGATGGCAGGAAAAAAATACTGTGGGACAGCAAAAAGCGACGCAATGAGGCACTCGACTGCTTCGTTTATGCGCTGGCGGCGCTGCGCATCAGTATTTCCCGCTGGCAGCTGGATCTCAGTGCGCTGCTGGCGAGCCTGCAGGAAGAGGATGGTGCAGCAACCAACAAGAAAACACTGGCAGATTACGCCCGTGCCTTATCCGGAGAGGATGAATGACGCGACAGGAAGAACTTGCCGCTGCCCGTGCGGCACTGCATGACCTGATGACAGGTAAACGGGTGGCAACAGTACAGAAAGACGGACGAAGGGTGGAGTTTACGGCCACTTCCGTGTCTGACCTGAAAAAATATATTGCAGAGCTGGAAGTGCAGACCGGCATGACACAGCGACGCAGGGGACCTGCAGGATTTTATGTATGAAAACGCCCACCATTCCCACCCTTCTGGGGCCGGACGGCATGACATCGCTGCGCGAATATGCCGGTTATCACGGCGGTGGCAGCGGATTTGGAGGGCAGTTGCGGTCGTGGAACCCACCGAGTGAAAGTGTGGATGCAGCCCTGTTGCCCAACTTTACCCGTGGCAATGCCCGCGCAGACGATCTGGTACGCAATAACGGCTATGCCGCCAACGCCATCCAGCTGCATCAGGATCATATCGTCGGGTCTTTTTTCCGGCTCAGTCATCGCCCAAGCTGGCGCTATCTGGGCATCGGGGAGGAAGAAGCCCGTGCCTTTTCCCGCGAGGTTGAAGCGGCATGGAAAGAGTTTGCCGAGGATGACTGCTGCTGCATTGACGTTGAGCGAAAACGCACGTTTACCATGATGATTCGGGAAGGTGTGGCCATGCACGCCTTTAACGGTGAACTGTTCGTTCAGGCCACCTGGGATACCAGTTCGTCGCGGCTTTTCCGGACACAGTTCCGGATGGTCAGCCCGAAGCGCATCAGCAACCCGAACAATACCGGCGACAGCCGGAACTGCCGTGCCGGTGTGCAGATTAATGACAGCGGTGCGGCGCTGGGATATTACGTCAGCGAGGACGGGTATCCTGGCTGGATGCCGCAGAAATGGACATGGATACCCCGTGAGTTACCCGGCGGGCGCGCCTCGTTCATTCACGTTTTTGAACCCGTGGAGGACGGGCAGACTCGCGGTGCAAATGTGTTTTACAGCGTGATGGAGCAGATGAAGATGCTCGACACGCTGCAGAACACGCAGCTGCAGAGCGCCATTGTGAAGGCGATGTATGCCGCCACCATTGAGAGTGAGCTGGATACGCAGTCAGCGATGGATTTTATTCTGGGCGCGAACAGTCAGGAGCAGCGGGAAAGGCTGACCGGCTGGATTGGTGAAATTGCCGCGTATTACGCCGCAGCGCCGGTCCGGCTGGGAGGCGCAAAAGTACCGCACCTGATGCCGGGTGACTCACTGAACCTGCAGACGGCTCAGGATACGGATAACGGCTACTCCGTGTTTGAGCAGTCACTGCTGCGGTATATCGCTGCCGGGCTGGGTGTCTCGTATGAGCAGCTTTCCCGGAATTACGCCCAGATGAGCTACTCCACGGCACGGGCCAGTGCGAACGAGTCGTGGGCGTACTTTATGGGGCGGCGAAAATTCGTCGCATCCCGTCAGGCGAGCCAGATGTTTCTGTGCTGGCTGGAAGAGGCCATCGTTCGCCGCGTGGTGACGTTACCTTCAAAAGCGCGCTTCAGTTTTCAGGAAGCCCGCAGTGCCTGGGGGAACTGCGACTGGATAGGCTCCGGTCGTATGGCCATCGATGGTCTGAAAGAAGTTCAGGAAGCGGTGATGCTGATAGAAGCCGGACTGAGTACCTACGAGAAAGAGTGCGCAAAACGCGGTGACGACTATCAGGAAATTTTTGCCCAGCAGGTCCGTGAAACGATGGAGCGCCGTGCAGCCGGTCTTAAACCGCCCGCCTGGGCGGCTGCAGCATTTGAATCCGGGCTGCGACAATCAACAGAGGAGGAGAAGAGTGACAGCAGAGCTGCGTAATCTCCCGCATATTGCCAGCATGGCCTTTAATGAGCCGCTGATGCTTGAACCCGCCTATGCGCGGGTTTTCTTTTGTGCGCTTGCAGGCCAGCTTGGGATCAGCAGCCTGACGGATGCGGTGTCCGGCGACAGCCTGACTGCCCAGGAGGCACTCGCGACGCTGGCATTATCCGGTGATGATGACGGACCACGACAGGCCCGCAGTTATCAGGTCATGAACGGCATCGCCGTGCTGCCGGTGTCCGGCACGCTGGTCAGCCGGACGCGGGCGCTGCAGCCGTACTCGGGGATGACCGGTTACAACGGCATTATCGCCCGTCTGCAACAGGCTGCCAGCGATCCGATGGTGGACGGCATTCTGCTCGATATGGACACGCCCGGCGGGATGGTGGCGGGGGCATTTGACTGCGCTGACATCATCGCCCGTGTGCGTGACATAAAACCGGTATGGGCGCTTGCCAACGACATGAACTGCAGTGCAGGTCAGTTGCTTGCCAGTGCCGCCTCCCGGCGTCTGGTCACGCAGACCGCCCGGACAGGCTCCATCGGCGTCATGATGGCTCACAGTAATTACGGTGCTGCGCTGGAGAAACAGGGTGTGGAAATCACGCTGATTTACAGCGGCAGCCATAAGGTGGATGGCAACCCCTACAGCCATCTTCCGGATGACGTCCGGGAGACACTGCAGTCCCGGATGGACGCAACCCGCCAGATGTTTGCGCAGAAGGTGTCCAGCAAAAGGCCAGGAACCGTAAAAAGGCCGCGTTGCTGGCGTTTTTCCATAGGCTCCGCCCCCCTGACGAGCATCACAAAAATCGACGCTCAAGTCAGAGGTGGCGAAACCCGACAGGACTATAAAGATACCAGGCGTTTCCCCCTGGAAGCTCCCTCGTGCGCTCTCCTGTTCCGACCCTGCCGCTTACCGGATACCTGTCCGCCTTTCTCCCTTCGGGAAGCGTGGCGCTTTCTCATAGCTCACGCTGTAGGTATCTCAGTTCGGTGTAGGTCGTTCGCTCCAAGCTGGGCTGTGTGCACGAACCCCCCGTTCAGCCCGACCGCTGCGCCTTATCCGGTAACTATCGTCTTGAGTCCAACCCGGTAAGACACGACTTATCGCCACTGGCAGCAGCCACTGGTAACAGGATTAGCAGAGCGAGGTATGTAGGCGGTGCTACAGAGTTCTTGAAGTGGTGGCCTAACTACGGCTACACTAGAAGGACAGTATTTGGTATCTGCGCTCTGCTGAAGCCAGTTACCTTCGGAAAAAGAGTTGGTAGCTCTTGATCCGGCAAACAAACCACCGCTGGTAGCGGTGGTTTTTTTGTTTGCAAGCAGCAGATTACGCGCAGAAAAAAAGGATCTCAAGAAGATCCTTTGATCTTTTCTACGGGGTCTGACGCTCAGTGGAACGAAAACTCACGTTAAGGGATTTTGGTCATGAGATTATCAAAAAGGATCTTCACCTAGATCCTTTTAAATTAAAAATGAAGTTTTAAATCAATCTAAAGTATATATGAGTAAACTTGGTCTGACAGTTACCAATGCTTAATCAGTGAGGCACCTATCTCAGCGATCTGTCTATTTCGTTCATCCATAGTTGCCTGACTCCCCGTCGTGTAGATAACTACGATACGGGAGGGCTTACCATCTGGCCCCAGTGCTGCAATGATACCGCGAGACCCACGCTCACCGGCTCCAGATTTATCAGCAATAAACCAGCCAGCCGGAAGGGCCGAGCGCAGAAGTGGTCCTGCAACTTTATCCGCCTCCATCCAGTCTATTAATTGTTGCCGGGAAGCTAGAGTAAGTAGTTCGCCAGTTAATAGTTTGCGCAACGTTGTTGCCATTGCTACAGGCATCGTGGTGTCACGCTCGTCGTTTGGTATGGCTTCATTCAGCTCCGGTTCCCAACGATCAAGGCGAGTTACATGATCCCCCATGTTGTGCAAAAAAGCGGTTAGCTCCTTCGGTCCTCCGATCGTTGTCAGAAGTAAGTTGGCCGCAGTGTTATCACTCATGGTTATGGCAGCACTGCATAATTCTCTTACTGTCATGCCATCCGTAAGATGCTTTTCTGTGACTGGTGAGTACTCAACCAAGTCATTCTGAGAATAGTGTATGCGGCGACCGAGTTGCTCTTGCCCGGCGTCAATACGGGATAATACCGCGCCACATAGCAGAACTTTAAAAGTGCTCATCATTGGAAAACGTTCTTCGGGGCGAAAACTCTCAAGGATCTTACCGCTGTTGAGATCCAGTTCGATGTAACCCACTCGTGCACCCAACTGATCTTCAGCATCTTTTACTTTCACCAGCGTTTCTGGGTGAGCAAAAACAGGAAGGCAAAATGCCGCAAAAAAGGGAATAAGGGCGACACGGAAATGTTGAATACTCATACTCTTCCTTTTTCAATATTATTGAAGCATTTATCAGGGTTATTGTCTCATGAGCGGATACATATTTGAATGTATTTAGAAAAATAAACAAATAGGGGTTCCGCGCACATTTCCCCGTACGGATCCAAGCGGCCTGGTGCCGCGCGGCAGCATGATCCTCGACACTGACTACATAACCGAGGATGGAAAGCCTGTCATAAGAATTTTCAAGAAGGAAAACGGCGAGTTTAAGATTGAGTACGACCGGACTTTTGAACCCTACTTCTACGCCCTCCTGAAGGACGATTCTGCCATTGAGGAAGTCAAGAAGATAACCGCCGAGAGGCACGGGACGGTTGTAACGGTTAAGCGGGTTGAAAAGGTTCAGAAGAAGTTCCTCGGGAGACCAGTTGAGGTCTGGAAACTCTACTTTACTCATCCGCAGGACGTCCCAGCGATAAGGGACAAGATACGAGAGCATCCAGCAGTTATTGACATCTACGAGTACGACATACCCTTCGCCAAGCGCTACCTCATAGACAAGGGATTAGTGCCAATGGAAGGCGACGAGGAGCTGAAAATGCTCGCCTTCGCGATTGCGACTCTCTACCATGAGGGCGAGGAGTTCGCCGAGGGGCCAATCCTTATGATAAGCTACGCCGACGAGGAAGGGGCCAGGGTGATAACTTGGAAGAACGTGGATCTCCCCTACGTTGACGTCGTCTCGACGGAGAGGGAGATGATAAAGCGCTTCCTCCGTGTTGTGAAGGAGAAAGACCCGGACGTTCTCATAACCTACAACGGCGACAACTTCGACTTCGCCTATCTGAAAAAGCGCTGTGAAAAGCTCGGAATAAACTTCGCCCTCGGAAGGGATGGAAGCGAGCCGAAGATTCAGAGGATGGGCGACAGGTTTGCCGTCGAAGTGAAGGGACGGATACACTTCGATCTCTATCCTGTGATAAGACGGACGATAAACCTGCCCACATACACGCTTGAGGCCGTTTATGAAGCCGTCTTCGGTCAGCCGAAGGAGAAGGTTTACGCTGAGGAAATAACCACAGCCTGGGAAACCGGCGAGAACCTTGAGAGAGTCGCCCGCTACTCGATGGAAGATGCGAAGGTCACATACGAGCTTGGGAAGGAGTTCCTTCCGATGGAGGCCCAGCTTTCTCGCTTAATCGGCCAGTCCCTCTGGGACGTCTCCCGCTCCAGCACTGGCAACCTCGTTGAGTGGTTCCTCCTCAGGAAGGCCTATGAGAGGAATGAGCTGGCCCCGAACAAGCCCGATGAAAAGGAGCTGGCCAGAAGACGGCAGAGCTATGAAGGAGGCTATGTAAAAGAGCCCGAGAGAGGGTTGTGGGAGAACATAGTGTACCTAGATTTTAGATCCCTGTACCCCTCAATCATCATCACCCACAACGTCTCGCCGGATACGCTCAACAGAGAAGGATGCAAGGAATATGACGTTGCCCCACAGGTCGGCCACCGCTTCTGCAAGGACTTCCCAGGATTTATCCCGAGCCTGCTTGGAGACCTCCTAGAGGAGAGGCAGAAGATAAAGAAGAAGATGAAGGCCACGATTGACCCGATCGAGAGGAAGCTCCTCGATTACAGGCAGAGGCGCATCAAGATCCTGGCAAACAGCTACTACGGTTACTACGGCTATGCAAGGGCGCGCTGGTACTGCAAGGAGTGTGCAGAGAGCGTAACGGCCTGGGGAAGGGAGTACATAACGATGACCATCAAGGAGATAGAGGAAAAGTACGGCTTTAAGGTAATCTACAGCGACACCGACGGATTTTTTGCCACAATACCTGGAGCCGATGCTGAAACCGTCAAAAAGAAGGCTATGGAGTTCCTCAAGTATATCAACGCCAAACTTCCGGGCGCGCTTGAGCTCGAGTACGAGGGCTTCTACAAACGCGGCTTCTTCGTCACGAAGAAGAAGTATGCGGTGATAGACGAGGAAGGCAAGATAACAACGCGCGGACTTGAGATTGTGAGGCGTGACTGGAGCGAGATAGCGAAAGAGACGCAGGCGAGGGTTCTTGAAGCTTTGCTAAAGGACGGTGACGTCGAGAAGGCCGTGAGGATAGTCAAAGAAGTTACCGAAAAGCTGAGCAAGTACGAGGTTCCGCCGGAGAAGCTGGTGATCCACATACAGATAACGAGGGATTTAAAGGACTACAAGGCAACCGGTCCCCACGTTGCCGTTGCCAAGAGGTTGGCCGCGAGAGGAGTCAAAATACGCCCTGGAACGGTGATAAGCTACATCGTGCTCAAGGGCTCTGGGAGGATAGGCGACAGGGCGATACCGTTCGACGAGTTCGACCCGACGAAGCACAAGTACGACGCCGAGTACTACATTGAGAACCAGGTTCTCCCAGCCGTTGAGAGAATTCTGAGAGCCTTCGGTTACCGCAAGGAAGACCTGCGCTACCAGAAGACGAGACAGGTTGGTTTGAGTGCTTGGCTGAAGCCGAAGGGAACTTGAGTCGACCTGCAGCCAAGCTTAATTAGCTGAGCTTGGACTCCTGTTGATAGATCCAGTAATGACCTCAGAACTCCATCTGGATTTGTTCAGAACGCTCGGTTGCCGCCGGGCGTTTTTTATTGGTGAGAATCCAAGCTAGCTTGGCGAGATTTTCAGGAGCTAAGGAAGCTAAAATGGAGAAAAAAATCACTGGATATACCACCGTTGATATATCCCAATGGCATCGTAAAGAACATTTTGAGGCATTTCAGTCAGTTGCTCAATGTACCTATAACCAGACCGTTCAGCTGGATATTACGGCCTTTTTAAAGACCGTAAAGAAAAATAAGCACAAGTTTTATCCGGCCTTTATTCACATTCTTGCCCGCCTGATGAATGCTCATCCGGAATTTCGTATGGCAATGAAAGACGGTGAGCTGGTGATATGGGATAGTGTTCACCCTTGTTACACCGTTTTCCATGAGCAAACTGAAACGTTTTCATCGCTCTGGAGTGAATACCACGACGATTTCCGGCAGTTTCTACACATATATTCGCAAGATGTGGCGTGTTACGGTGAAAACCTGGCCTATTTCCCTAAAGGGTTTATTGAGAATATGTTTTTCGTCTCAGCCAATCCCTGGGTGAGTTTCACCAGTTTTGATTTAAACGTGGCCAATATGGACAACTTCTTCGCCCCCGTTTTCACCATGGGCAAATATTATACGCAAGGCGACAAGGTGCTGATGCCGCTGGCGATTCAGGTTCATCATGCCGTTTGTGATGGCTTCCATGTCGGCAGAATGCTTAATGAATTACAACAGTACTGCGATGAGTGGCAGGGCGGGGCGTAATTTTTTTAAGGCAGTTATTGGTGCCCTTAAACGCCTGGGGTAATGACTCTCTAGCTTACGCGCCCTGTAGCGGCGCATTAAGCGCGGCGGGTGTGGTGGTTACGCGCAGCGTGACCGCTACACTTGCCAGCGCCCTAGCGCCCGCTCCTTTCGCTTTCTTCCCTTCCTTTCTCGCCACGTTCGCCGGCTTTCCCCGTCAAGCTCTAAATCGGGGGCTCCCTTTAGGGTTCCGATTTAGTGCTTTACGGCACCTCGACCCCAAAAAACTTGATTTGGGTGATGGTTCACGTAGTGGGCCATCGCCCTGATAGACGGTTTTTCGCCCTTTGACGTTGGAGTCCACGTTCTTTAATAGTGGACTCTTGTTCCAAACTGGAACAACACTCAACCCTATCTCGGGCTATTCTTTTGATTTATAAGGGA |

Table S2. Primers amplified custom-designed DNA fragments

| Primer Name | Primer Sequence (5’-3’) |
| --- | --- |
| PF1 | TGCGATGAGTGGCAGGGCGGGGCGTAATTTTTTTAAGGCAGTTATTGGTGCC |
| PF2 | CCAGCAAAAGGCCAGGAACCG |
| PF3 | CAAATAGGGGTTCCGCGCACATTTCCCCGTACGGATCCAAGCGGCCTG |
| PF4 | ATGGAGAAAAAAATCACTGGATATACCAC |
| PF5 | CGGCCTTTTTACGGTTCCTGGCCTTTTGCTGGACACCTTCTGCGCAAACATCT |
| PF6 | CGGGGAAATGTGCGCGGAACC |
| PF7 | GTGGTATATCCAGTGATTTTTTTCTCCATTTTAGCTTCCTTAGCTCCTGAAAAT |
| PF8 | TTACGCCCCGCCCTGCCAC |

Table S3. The sequence of staple strands in large rectangular DNA origami.

| Start | End | Sequence |
| --- | --- | --- |
| 0[167] | 1[167] | CTGGTTTATTGCTGATTATCATTGCAGACTTC |
| 2[239] | 1[255] | GCGTTCTGTAAAAAACGCCCGGCGTGATTTTT |
| 37[40] | 37[88] | CCCGTATCCCCAGCATCCGGACGTTCGCCAATTTTCGACGGCAGTTCCG |
| 28[87] | 28[56] | CTTCCCGAATCATTCCGGCGGGTATAGGTTTT |
| 25[168] | 25[215] | GCCGCATACACTTGACTTCCTCAATGGCAGAATCGTCGGATCTAAAAT |
| 10[87] | 10[56] | CACTTTCACTCGAGATCCAGCTGCCAGCGGGA |
| 1[128] | 0[144] | CGCATAGGAGCGTGGGTCTCGCGGAAATCTGG |
| 15[216] | 16[216] | GCCAGTTTTGACTCCTGGGACCGGTTGCCTGA |
| 20[127] | 19[127] | CGAGCAGACCAGCCCGGCAGCGATTGCTCATACGAGACACATGCCGTC |
| 20[239] | 19[255] | TCTTTGACTACTTGCTCAGCTTTTACGTTTCA |
| 6[55] | 7[39] | GATAAAATATCATCATCAAAATGCTCTATAAAAACCGACACGGGAGAG |
| 17[16] | 14[16] | GTTCATCCATTCACTCAGCAACCCACAAAATGCCGCAGCCGCGAAGCC |
| 35[168] | 35[215] | TTGGGTGCACGACAATAACCCTGATAAATGCTTCAATGCCTCTCCTCT |
| 25[128] | 24[128] | CGGTTTTAGTTCGTGCACACAGCCTCGGGCTGAACGGGGGTGTCACGC |
| 35[216] | 36[216] | AGGAGCGCTCTCTGCAGCCGTAGTAACTTCTT |
| 19[40] | 19[87] | TCTTTGTCGCCCAGTGTGAAGCGGTTATAAATCTGCTCCAGCTTGGGC |
| 35[256] | 36[240] | CAGCACCTATGAACCTGAATCGCCAGCGCGCC |
| 12[239] | 11[255] | AACGGTATTGCTTCGTCGGGTCGAAAGGCCGG |
| 23[216] | 24[216] | TCGGGCTTTCGCTATCGCGCGTTGTTATACAC |
| 7[216] | 8[216] | CTCTCAACCAACCTGTGTAACCGAAGGTCGCA |
| 19[216] | 20[216] | GCTTGGCGGAACCTCGTATCCTCACGGGTCTT |
| 13[128] | 12[128] | CAGCGTGCGAGATCCTTTTTTTCTATCAAAGGATCTTCTTCGGACACC |
| 0[199] | 0[168] | GTCCGTCTTATCACAGGATAGCTTCCGGCTGG |
| 32[239] | 31[255] | ATTACCTTAAAAATCCGTCGGTGTAGTTGTCC |
| 17[40] | 17[87] | CCCGCAATGCGGGTTATTCGGGAAGTGAACGGCACCGACCCGACGATA |
| 16[239] | 15[255] | GCAACGTGCTCGCGGCCAACCTCTACCGTCTT |
| 27[88] | 28[88] | CGTGCGCTGCTCCTGACTGCGTATCCACACAC |
| 10[239] | 9[255] | TTCTCAATCTCTCAACGGCTGGGATACGGTCT |
| 3[168] | 3[215] | GATGGAGGCGGTCTGAATCTTCGGCTCGCTTCCATCCACCTTCTCCTT |
| 32[15] | 29[15] | ACGGGCATGGCAGAGTCATAAAGCACCATTTTATGACGGCCAGCTTTT |
| 33[128] | 32[128] | TAATTACTAGCGTCGATTTTTGTGCGCCACCTCTGACTTGGTGAGCCA |
| 24[127] | 23[127] | ACACGGGCGTACTTTTGCGCCTCCCCCGGCATCAGGTGCGGATGATGT |
| 35[40] | 35[87] | GACAATCTGCACACGGTCAGGAACGGGCGCTGAATAAGGTTGCTGATG |
| 5[128] | 4[128] | CGGACACCGACAGATCGCTGAGATATGGATGAACGAAATAGCATCCGT |
| 36[167] | 37[167] | TGAAGCCTTTTGCTGGTCCATCCGGGAAGAAA |
| 22[239] | 21[255] | AGAACCCTTCACCGTCCTTTAGCAATCGTCGT |
| 36[215] | 36[168] | CTTTATCTTCTAATATTGAAAAAGGAAGAGTATGAGTGTAAAAGATGC |
| 7[88] | 8[88] | CGCCGCACCGCGTTTTTTCTATCAGCAGCAAC |
| 23[256] | 24[240] | ATATATGTACCGTAACACGCCACATCACAATC |
| 22[87] | 22[56] | GGGAAAAGGCACTTACGTGGCATGCTGGCCAC |
| 2[15] | 0[8] | TATCACGACGGTGCTTTTTGCCATGGTGAGCGTGTTATCC |
| 21[256] | 22[240] | GGTATTCAGTAGAAACTGCCGGAAAAGCTTCA |
| 33[256] | 34[240] | CCATGGTGTGTCGCCTTGCGTATATTATGTAC |
| 19[88] | 20[88] | GATGATCCGGGAAAGCATACCGCAGCACGATG |
| 11[88] | 12[88] | GCTGCTCCAGTCGCAGGAAGCGCGCTTTTGCC |
| 35[88] | 36[88] | CGCTTAGGCGCGCCCGCTGCGGCATCCTCGCC |
| 33[40] | 33[87] | ACGCTGTCCAGCGCATTTCGTAGCGGTCCAGCTGGGAAACTGTGTCCG |
| 28[239] | 27[255] | CCCGGAAGCCCTCGTACTCGAGCTGAAAAACA |
| 2[87] | 2[56] | TTCATACATAAGTGGCCGTAAACTCCACCCTT |
| 19[168] | 19[215] | CTGCCATAACCGGATGCTCTCGTATCTTGTCCCTTATCTTTTCATCGG |
| 27[216] | 28[216] | GATGAGTTTGTAGAAGTTTGGCGTTGAGAGCG |
| 13[216] | 14[216] | ACTGGCTATCCTCCCATCCAGGGCGTATGCTG |
| 20[55] | 21[39] | TTTCGCATTTGAGGCCAAACGCAGTTCCGGAAAGAATACTGGCGGGGT |
| 17[128] | 16[128] | GGGCGATATAACTGGCTTCAGCAGACTCTTTTTCCGAAGGATGCCGTT |
| 5[168] | 5[215] | ACTCTAGCTTCCTTTTCACAGCGCTTTTTCAGATAGGTTTCCCAGGCT |
| 0[79] | 0[40] | CATGCCGGTCTGCACTTCCAGCTCTGCAAGCCACGCCCAT |
| 11[16] | 8[16] | TTCCTGGTAAAGGGCAGTGTTTCCATGTAGTCGCTGCCCACACATTCA |
| 8[15] | 5[15] | CCTCACGGAGCAGCATTTTGGAATGTAGGCATAAACACCCAACCAGAT |
| 18[215] | 18[168] | GGCCAGCTCATCGTACTCGTAGATGTCAATAACTGCTATGAGTGATAA |
| 26[239] | 25[255] | TTCGTGACTCGTCTATCACCGCATAATAGGCC |
| 39[232] | 39[263] | CATCGCAGTACTGTTGTAATTCATTAAGCATT |
| 37[89] | 39[95] | GCTGAGCCAGGATACACCGCTGTCATTAATCAGCGAAAA |
| 10[127] | 9[127] | ACTGCGGGCATCGATGGCCATACGTGAACTTCTTTCAGACCCTGTCGT |
| 26[127] | 25[127] | TGGCAAGCCACCAATCCAGCCGGTTAATACGCGGCAATTTGCCCATAC |
| 22[215] | 22[168] | TTACATAGCCTCTCCCGAGGAACTTCTTCTGAACCTTAGTCACAGAAA |
| 23[168] | 23[215] | GAGTACTCACCTTCAACCCGCTTAACCGTTACAACCGACAACCCTCTC |
| 7[40] | 7[87] | CCTTCCTGTTCGGCACGGGCGTAATCTGCCAGTGTTTTCCGCTGCCAC |
| 29[256] | 30[240] | ACTCACCCCACGTTTAAATCAAAATTTTGACG |
| 38[63] | 39[79] | ACCGAGCGGAAACGCCTTAAACCGGAAAATTT |
| 2[127] | 1[127] | CACAAAAGCACTCTTCTCCTCCTCTACGCAGCTCTGCTGTAAAACCCG |
| 0[39] | 1[39] | TAGTGAAACGCTTCATACCACGGGGCCCGCCC |
| 1[88] | 2[88] | TCCCCATGCGGGAGATTGTTGATTGTCGCGTT |
| 19[128] | 18[128] | CACCATCGAGTTAGGCCACCACTTCTTCTAGTGTAGCCGTGATCGCTG |
| 4[127] | 3[127] | CAGGCTGCCCCAGGCGGGCGGTTTTCAAATGCTGCAGCCGTGATCCCA |
| 22[55] | 23[39] | CGGCTGATAAAACACGCTGGAAATTTCACCTGGTTTCCACCACGCATT |
| 3[256] | 4[240] | ATATATCAAATGTTCTTTACGATGTATCAACA |
| 34[215] | 34[168] | CAAGCAGGCTCCATTCAAATATGTATCCGCTCATGAGAGTGGGTTACA |
| 12[87] | 12[56] | ACGGGTAAAGTACGAAGCAGTCGAGTGCCTCA |
| 22[127] | 21[127] | CCCCGCCATATCCGTATCCTGAGCAACACGGAGTAGCCGTCCATCCCG |
| 27[168] | 27[215] | CCGTATTGACGCAAAAGTCCGGTCGTACTCAATCTTAACGTTGTGGGT |
| 31[88] | 32[88] | CCTGAGCGTGTTCTGCGCTGTAAAACAGCCGC |
| 13[168] | 13[215] | ATCATGTAACTGGCCCCTCGGCGAACTCCTCGCCCTCGTCCCAGAGGG |
| 26[87] | 26[56] | CGCTCAACGTCGGTCAATCCCGCCAGTATCCC |
| 8[87] | 8[56] | TGCCCTCCAAATCTTGTTGGTTGCTGCACCAT |
| 16[87] | 16[56] | GCAGCTGGATGCGCTTCGGTCAGATCAAAAAT |
| 25[40] | 25[87] | TCCAGCTCCTGTTTCGAGCGTTCATACACAATGGTCGAATGCAGCAGC |
| 26[167] | 27[167] | CAACTGAACGACCTACAGAAAGCGCCATTATC |
| 12[127] | 11[127] | GGCAGCACGGGCTTCCTGAAAACTTTCCCCCAGGCACTGCGGCGATGC |
| 38[127] | 37[127] | TGTAGGGGAATATCCCAGCGCCGCCCGTCCTCGCTGACGTTTGCCATC |
| 31[256] | 32[240] | ATATTGGCAAAACGGGGGCGAAGACGCTGTAG |
| 16[215] | 16[168] | GGAGGAACCACTCCATTGGCACTAATCCCTTGTCTATGACAACGATCG |
| 5[16] | 2[16] | AAGGGTGTATCACCATCCGTCGGCATAGTCGGCTCAACGTCGACGGAA |
| 39[128] | 38[128] | CGCTTAATGCGCCGCTACAGGGCGCCCGGACGTCATCCGGAAGATGGC |
| 15[128] | 14[128] | GAGTACGGTACCAGCGGTGGTTTGACAAAAAAACCACCGCCTGCAGCG |
| 24[167] | 25[167] | TGACTAGGCGCAGCGGCAGCTTGGAGCCGGTC |
| 6[167] | 7[167] | CGAACTGATTAAGCATACTTTAGATTGAACAA |
| 25[88] | 26[88] | AGTCAGCGCTGCGGCGCAGCCTTTCCCGTTTT |
| 23[128] | 22[128] | CAGCGCAGTGGACTCAAGACGATATCGTGTCTTACCGGGTTCAAATGC |
| 30[239] | 29[255] | GTTTCAGCAACTCCATAGCCTTCTCTGGTGAA |
| 5[256] | 6[240] | TGCCTCAAGGTACATTGAGCAACTTTCCCTTC |
| 32[215] | 32[168] | TTGCAGAAGCGATCCGTACGGGGAAATGTGCGCGGAAGTAAGATCCTT |
| 29[216] | 30[216] | CTGTTTATACTTGAGGATCGGCTCCAGGCAAC |
| 4[87] | 4[40] | CCGGCCCCAGATACCTGTCATCAGGTCATGCAGTGCCAACCCGCCACA |
| 7[256] | 8[240] | TGGTTATAGCCGTAATATCCAGCTGCAGGTCT |
| 1[200] | 2[216] | TTCACATAAACGGCCTCAAGCATTCTCACCAAAACAAATCCAGGACCG |
| 18[55] | 19[39] | GGCGTAAATTTAAGATACTGCTTTGTTCATGGTGTCGGTTTTCGCGTC |
| 34[87] | 34[56] | TGACCATCCGGGTCGATACCGGCGGTCAGGTA |
| 37[216] | 39[231] | TCATCCGTAGTAGCTGATCGAGGAGCTTCCTCGCCCCGCCCTGCCACT |
| 24[215] | 24[168] | TATGTTCTCCCTCCCGTGCCTCTCGGCGGTTATCTTCTATTCTCAGAA |
| 39[32] | 38[48] | ATTAAGTTATGACGAAGAAGAACGTGGTGTTT |
| 12[55] | 13[39] | TTGCGGCCTCTCACTTTTGGCTTCGGGAGATAGTGGCTCCGGCGCTCA |
| 33[88] | 34[88] | GAAAACATTTGCACCGGTGAATGAACGCGGGC |
| 8[55] | 9[39] | CCTCTTCACCCAGGAACGTCGGCAGACTTCACTCGCATTCATGGCCTT |
| 14[167] | 15[167] | AACATGCTGCTTGCAATTTGCCGGATCCCGAA |
| 24[239] | 23[255] | TCAAGTCCTCGCTCCAGTCACGCCTCTTGCGA |
| 6[215] | 6[168] | GTTCTCGCCGGCGAAGTCGAAGTTGTCGCCGTTGTAGCTATTAACTGG |
| 39[8] | 37[15] | TCAGAGGGTATTTTAAGTCGTTTCCTTTCTTTGAAACGAC |
| 21[128] | 20[128] | CCGGGCGTTAATCCTGTTACCAGTACATACCTCGCTCTGCGTCCATAT |
| 39[96] | 39[127] | CCCGAAATCGGCAAAAACCACCACACCCGCCG |
| 3[216] | 4[216] | CGGCTATGGAGTTCTGAGCTCAGCTAATATTT |
| 16[55] | 17[39] | ATCCGGGCAGGCAACATGAAACTGTTCGATATAGCGCGGCTGCTCCTC |
| 8[127] | 7[127] | CGGATAATAGGTACTCAGTCCGGCGCGCACTCTTTCTCGTGCCAGCGT |
| 38[175] | 39[159] | TTTTTGCTCACCCCTGCAGTGTCTCGTAAGCT |
| 37[168] | 37[215] | CGCTGGTGAAAATTCAACATTTCCGTGTCGCCCTTATAATCGTGGCCT |
| 10[167] | 11[167] | ACGACATCTCATGACCAGCGTCAGACCAACCG |
| 30[127] | 29[127] | TGCGTGACTCGCCTTCACAATGGCATGGTGGCGGCATACACAGACGCC |
| 9[256] | 10[240] | TTAAAAAGTGTGCTTATTTTTCTTGAACCTGG |
| 28[215] | 28[168] | TATCCGGCGAGAACTCGCCGTTTTCCTTCTTGAAAATGCTATGTGGCG |
| 34[239] | 33[255] | TCCCTTCCTCCTTGATGGTCATCGATATTTGC |
| 11[168] | 11[215] | GAGCTGAATGACCAAGTTATCACCCTGGCCCCTTCCTGCTGGGCCTCC |
| 33[168] | 33[215] | TCTCAACAGCGCCCCTATTTGTTTATTTTTCTAAATAGGGATAAATCC |
| 4[167] | 5[167] | TAATAGTCAGGCAACTAGGTGCCTCACTACTT |
| 14[15] | 11[15] | CGGCGCGGCATTCAACGGCGGTCTGTAATTAGCATCCGCCCAGCGCCC |
| 14[215] | 14[168] | GAGCGGGAGACATGGTAGAGAGTCGCAATCGCGAAGGCTTTTTTGCAC |
| 15[168] | 15[215] | GGAGCTAACCGCGAGCATTTTCAGCTCCTCGTCGCCTTCAACGAGGTT |
| 30[87] | 30[56] | CAGTTCACCGTTGTTCATCGTCGTGGCGGCCC |
| 9[88] | 10[88] | CGACCTCACCGCTTCCACCGGAGCCTAATCCA |
| 14[55] | 15[39] | TTTCGGATTCACTGGCTGCACCTGAACAGTGATGTTAACCTGAACGCA |
| 32[167] | 33[167] | GAGAGCTGTCGGGTTTATGCTCGTCAGCTGGA |
| 1[168] | 1[199] | TGCGCTCGGCCAGATCGAAGTGTATCCGTCCC |
| 10[215] | 10[168] | ACTCCTTCCCATCAACGTAGGGGAGATCCACGTTCTTAGCCATACCAA |
| 31[40] | 31[87] | GGTGGCTCAATAATCTGCCGGTCAATCAGCCAGCTTTATCCCAGGTGG |
| 29[40] | 29[87] | CCAGAGAATGCTGGCCTCATCCACACGCAGCAGCGTCTAAAGGCGTGC |
| 0[143] | 0[112] | AGCCGGTGCGGGTTCAAGCATCAGCGGCTCAT |
| 11[216] | 12[216] | ATCGGCGTCGTACTTGCGCCCTGTCGCCCGAT |
| 31[216] | 32[216] | GTGGGGTATTGTGGCAAAAGCCGTACTAGTCC |
| 24[87] | 24[56] | GGCAAACTCTTGGGATCACCCGGAACAGCCCA |
| 7[128] | 6[128] | CGCGAGTGAAGTTTACTCATATATTGGTAACTGTCAGACCCCTCCTGG |
| 18[127] | 17[127] | GCAGCCTGCCGTGGAGTAGCTCATTTCGCACTGGCCCGTGTTGCAGAC |
| 31[168] | 31[215] | GCCCCGAAGAACTGCCGCGCGGCACCAGGCCGCTTGGGTGGCCGACCT |
| 1[256] | 2[240] | TTCTCCATACGGTGGTATATCCAGGCAACCGA |
| 39[80] | 38[64] | TCATAAATTGCACACCGGCCCTCCCACGTCTC |
| 36[127] | 35[127] | CTGTAAATGTATCCATGTCCATTTCCGGGTAACTCACGGGCAGCGTGA |
| 36[239] | 35[255] | CTTGCATACACTCCTTGCAGTACCAGCGGCAT |
| 21[88] | 22[88] | CTCCCGTGACTGCTCACGTCTGCAGGTCTCGC |
| 23[16] | 20[16] | AGAATGCGCGATCCGCGACAGCACCAGGGGGAGCCCGTCGAACGCCGC |
| 16[167] | 17[167] | GAGGAAAGAGCTACCAAGCGCAGATACCGGCC |
| 2[215] | 2[168] | AAGACGGCTTCTTCGACGGCAAACCTGTCGCCCATCCATAAAGTTGCA |
| 39[208] | 38[192] | AAAATTACTCGATCGGGTCTCCCTTTTTTGCG |
| 8[167] | 9[167] | ATGGCATTTAAAACTTCCTTTTTGATAGAGCG |
| 12[167] | 13[167] | TTGGGCCGTAGAAAAGGCGCGTAATCTGGGGG |
| 20[167] | 21[167] | TTATGTAGCACCGCCTGGCTGCTGCCACTTAC |
| 2[167] | 3[167] | GGACCCACTGGGGCCACACGACGGGGAGACTG |
| 38[47] | 39[31] | ACGAAGGTTTTACTTTCAGCACCTATAAAAAC |
| 34[127] | 33[127] | CTCCAGCGCCACGGGTTCAAAAACCGAGTCTGCCCGTCCTCAGCACCG |
| 15[88] | 16[88] | ATAGCACATCTGGCTCCATAAAGTACGCTGAT |
| 28[167] | 29[167] | CGGTACGCTTCCCGAACAGGGTCGGAAGCACT |
| 30[55] | 31[39] | ATAATCTCTTCACCGGATGACCCTTTCGGCATACCTCATTACCTGTCA |
| 17[256] | 18[240] | AACACTATATGGAAAACGGTGTAATCTGTATG |
| 11[128] | 10[128] | CGTTCATGAGTTTTCGTTCCACTGAAAATCCCTTAACGTGACCTGATA |
| 18[87] | 18[56] | CCGGAAAAAAGGGAAGCGGTTCATCCCCTTCC |
| 8[215] | 8[168] | TCTTCCATCGACTTCACAACACGGAGGAAGCGCTTTATGCCTGTAGCA |
| 9[168] | 9[215] | TGACACCACGATCATCTCCCTCTCCGTCGAGACGACGAGCTCGTATGT |
| 19[256] | 20[240] | GTTTGCTCCTCCAGAGCGATGAAACGGTAACT |
| 9[216] | 10[216] | GACCTCTCTCAGAATTGTAGTACTCGGAAGGA |
| 27[128] | 26[128] | CTGCAGTTTACAGCGTGAGCTATGACCGAACTGAGATACCCATGTCGT |
| 5[88] | 6[88] | GTCATCACGGCGCTCCCTGATAGTCGTTGATA |
| 22[167] | 23[167] | AGCATGTGGCGATAAGGTTACCGGATATGGTT |
| 4[239] | 3[255] | GGAGTCCAAGGTCATTACTGGATCCCATTGGG |
| 14[127] | 13[127] | CCCGCGTCCGATGGCCTCTTCCAGGTCACCACGCGGCGAACGGCTGAC |
| 27[40] | 27[87] | TCTTTTCGCAGATACGGGATATCGACATTTCTGCACCATCATGGTAAA |
| 6[239] | 5[255] | GGCTTCAGTGCAGGTCGACTCAAGGACTGAAA |
| 0[111] | 0[80] | TAAAGGCCATGCTGGCAATTGCGTCGCTGTGT |
| 27[256] | 28[240] | TATTCTCAAGGGATTGGCTGAGACCAAGCGCG |
| 20[15] | 17[15] | TGCACCGATTTCAGGAAATCAACACTTTGATGATATCCCGCGGTATCA |
| 9[40] | 9[87] | CAATACGCTTGTCCTGCAGGCTCGCCAGCAGCGCACTGGTGGGTTCCA |
| 21[216] | 22[216] | CTGCCCCTTCTCGACGCGCCTGCGTCTCTCTT |
| 29[168] | 29[215] | TTTAAAGTTCTTCTTATGACAGGCTTTCCATCCTCGGTGCATCCTTCT |
| 13[256] | 14[240] | ATCAGGCGATACGAAATTCCGGATTGTAGCTT |
| 26[215] | 26[168] | GAGGGGTACAGCTTCAGGAGGGCGTAGAAGTAGGGTTCCGGGCAAGAG |
| 11[40] | 11[87] | GCCACGGATGGTGATGCGCAGCGCCGCCAGCGCATAATGGGCAACAGG |
| 37[256] | 38[240] | ACGGCATGCTGCCGACATGGAAGCTGCGCCTC |
| 35[128] | 34[128] | TTTCCACAGCAACGCGGCCTTTTTCTATGGAAAAACGCCACCCTGTTT |
| 17[168] | 17[215] | AACTTACTTCTGAGGTAGCGCTTGGCGAAGGGTATGTTCCTCTCATAG |
| 20[87] | 20[56] | CCCAGATAGCGCTTTCGCGGTATCCGTACCGA |
| 26[55] | 27[39] | AGCAGATATAACGGGCATCAGAAGTCGATGGCGCCTCGCTGGCCCCGG |
| 20[215] | 20[168] | CTGGCCAGCTCCGCTGGGACGTCCTGCGGATGAGTAACAGTAAGAGAA |
| 23[88] | 24[88] | TCAACTCAGTGAGTCACAGCCGGACCGTCCTC |
| 28[127] | 27[127] | AAGCAACTTAAAATCCATCGCTGACTGTTCGCGCCCAGAAGACCTGCA |
| 12[215] | 12[168] | TAAGCGAGAAACGTCGGCGTAGCTTATCATAAGGATTCGCCTTGATCG |
| 15[256] | 16[240] | TCATTGCCCCCATATCACCAGCTCTGGCAACG |
| 14[239] | 13[255] | ATCACCGTGAGCCCTTGAGCACGAGAGCATTC |
| 37[16] | 35[15] | AGAGGCCAACAAAAACAGAGAAAGCAGCTGCT |
| 3[40] | 3[87] | TTTCACGGTAGGTCTTTCTGTACTGTTGCCACCCGTTAGGGTGGGAAT |
| 29[88] | 30[88] | ATGGCGCTCACTCTCAGCTCTGCAGCTACGAA |
| 17[216] | 18[216] | GCCTTCCTTGTAGTCCCAGCTTCTCCGTTCGG |
| 36[55] | 37[39] | ACTTCTTTCGTTTTCATCCAGTCATTCCACGGAAAAGCCTCGCGTTTT |
| 17[88] | 18[88] | TGATCCCCACGACTCGCTGGGCGTAATCTGAG |
| 18[167] | 19[167] | CACTGCAAATACTGTCCAAGAACTCTGCAGTG |
| 15[40] | 15[87] | TAAAATGCGGGACCTGCTCTTCAGCAGTCAGCTGCTGGCGTTGGCGGC |
| 14[87] | 14[56] | TTGCGTACCAGTTTTCCTGCCATCCACCCATT |
| 3[88] | 4[88] | GGTGGGCAGCCCGGATAAGACCGGCTGGCCGT |
| 24[55] | 25[39] | TGTTTCTGGCGGATGACGCAGTTCAGCGGAGTTGCGCACGCGTTAAAG |
| 34[55] | 35[39] | AGCCACCCAGGTGGTGAACGGACGGATACTCGACTTCCATTGTTCTTT |
| 1[40] | 1[87] | AGGCACCAGAATATATTTTTTCAGGTCAGACACGGAAAATCCTGCAGG |
| 11[256] | 12[240] | ATAAAACTGGCAAGAATGTGAATAACTCGTCG |
| 26[15] | 23[15] | TCAATAGTTTCCTGTGCGTCGGCCCTCTGGCATTCTTCAGGAAAGTAC |
| 30[215] | 30[168] | GTCATATTCCTTTATGTAGTCAGTGTCGAGGATCATGCGTTTTCCAAT |
| 28[55] | 29[39] | ATTGACATCACGGGTCCAGATCTGCCGCAGTTATTTCAGCATCGAAAA |
| 5[56] | 5[87] | GGCAGCGGCAAGTTCTTCCTGCGCGCAGCGAT |
| 7[168] | 7[215] | CGTTGCGCAAAGTTATGAGAACGTCCGGGTCTTTCTCGTAGCGGGCGA |
| 37[128] | 36[128] | CACCTTATCATCTGGCGGGTTGCGACACCTTCTGCGCAAAGGCTGCCG |
| 4[39] | 5[55] | TCCACAAGTTCTCGGCTGCGCTGCTTAGCAGCAAGTTCATCATGCACG |
| 38[239] | 37[255] | TGCCTGTATTTGCCAGGATCTTGACATCACAA |
| 21[168] | 21[215] | GGATGGCATGAAGTAGAGTTTCCAGACCTCAACTGGTCCTTCATAGCT |
| 3[128] | 2[128] | AGCTGGCCGTATCGTAGTTATCTAGATGGTAAGCCCTCCCTGCAAGCG |
| 18[239] | 17[255] | TGGATCACTTTAAATCCCTCGTTACAAGGGTG |
| 9[128] | 8[128] | GGTCCGTCGGATCTAGGTGAAGATCATTTTTAATTTAAAAATCATCAC |
| 33[216] | 34[216] | TGGGATTTCCTCTATCCCAGGCCGTTAGTCTC |
| 32[55] | 33[39] | ATACGATGTGAAAGGTCACACCTTGCCACCGCTTCTGAATGGTGCTGT |
| 30[167] | 31[167] | GATGACAGGAGAGCGCTCTTTATAGTCTTTTC |
| 6[87] | 6[56] | ACCGGCATATTTCGCGTCATTCATCCTCTCCG |
| 35[16] | 32[16] | TTTTGTTGCACCGAAAATGTCAGCTCCCTGTTCCTGCCAGCTCGCAGA |
| 4[215] | 4[168] | CCTCAGCGTAACTTCCGAGGGCGAAGTTTATTCCGAGCCGGCAACAAT |
| 23[40] | 23[87] | ATGCTCGCAGATTCCGTAGACGGATGCCCCTTTAATGTCCATGCCGCT |
| 25[216] | 26[216] | CTAGGTCTTGCCTTCCGAAGAAGCCGCTGATT |
| 29[128] | 28[128] | GGGAGGCGGGTATCCGGTAAGCGGGGGAGAAAGGCGGACAGCACTGGC |
| 32[127] | 31[127] | TCATGACGTCATCTGCTCCATCACAGCGTGTCGAGCATCTCCGATGGA |
| 38[191] | 39[207] | GCATTTTGAAGGGCACCAATAACTGCCTTAAA |
| 32[87] | 32[56] | GACGAACTGGTCCTCACCCGGCCCCCATCCCC |
| 10[55] | 11[39] | AATACACTTTGGCCAGACCGAATGGCCCGCTGATGCGGATTCTGTGGA |
| 25[256] | 26[240] | AGGTTTTCATAAACCCTTTAGGGAACTTCTTC |
| 8[239] | 7[255] | TCCTTGCGCTCGTCTTCTGGTAGCGAACGGTC |
| 13[40] | 13[87] | ATCTGACAGGTTCGCTTTTTGCTGTCCCACAGTATTTATCGTCTGCGC |
| 34[167] | 35[167] | TCGAAGGGGGCGGAGCACGGTTCCTGGATCAG |
| 6[127] | 5[127] | GCAGTCAGGCTGGGCAAAAATTTCATCGTTTCACGGACCTGCTGTCGC |
| 13[88] | 14[88] | GGGCATTGAAGGTAACCCAGCACAGAACGTTA |
| 0[231] | 0[200] | AGCTAGCTTGGGTGTATGTGGGCAGGTTTATC |
| 5[216] | 6[216] | GTGGTTTAAGCTTGGCCCAAGCACTCATCAAG |
| 29[16] | 26[16] | CGTTCTCACTTCAACCTCCCGGCGTCCTGGCTGGAGATCTGTTCGTAC |
| 2[55] | 3[39] | CGTCCTTTTTTGCCGCTTTACAGCGCCAGCAGGGGTTTTCATACGACT |
| 21[40] | 21[87] | CATCCGGCGTCGTAAGGTAAACCCCGTTTTTGTTTCGCGGGCTTCTTC |
| 31[128] | 30[128] | GCCTGTCCGGGGAAACGCCTGGTAACGAGGGAGCTTCCAGGGGCGGTC |
| 39[160] | 38[176] | AGAGAGTCATTACCCCAGGCGTTTCCTTCCTG |
| 36[87] | 36[56] | GGTATTGTTCGTGCTCTTTCCGCTCTGCCATC |
| 16[127] | 15[127] | GTAACCGGCGAATTTTCGCCGCCCGCCTGACGGGATGCGATCATCCCC |
| 0[263] | 0[232] | TTTAGCTTCCTTAGCTCCTGAAAATCTCGCCA |

Table S4. The sequence of staple strands in large triangular DNA origami.

| Start | End | Sequence |
| --- | --- | --- |
| 0[263] | 1[247] | CGCCGGGCGGTGAACGGGCTGTACCAATCTGC |
| 1[184] | 3[183] | GTTTACGACCGGACGTTCGCCAATGCCAGCTT |
| 1[200] | 1[183] | CGTTTTCCTGGCTCAATCTCTTCACCGGATGACGAAAACCAGCGTGGT |
| 1[216] | 3[215] | CTTTCGTTATGCTCTTTCCGCTCTACGCGCAT |
| 1[232] | 1[215] | GTCTTTGAGCTGTCCAGATGTGAAAGGTCACACTGTCAGGCGTATCCC |
| 1[248] | 0[264] | ACCCAGGTGTGTCCATATCGAGCACACCATCC |
| 1[280] | 3[279] | TCCACCATATGCCGTTCATGACCTTCACCGGA |
| 1[296] | 1[279] | TGGCAGCCGCGATGATGTCAGCGCAGTCAAATGCCCCCGCGAATGCCG |
| 1[312] | 3[311] | ACGGGCGATGACCAGCGTGCCGGACTGGGCAG |
| 1[328] | 1[311] | TTGTAACCGCGCCCATACCGGTTTTATGTCACGCACACGGTGTTGCAG |
| 1[336] | 11[175] | GGTCATCCCCGTGCCGCTGTAA |
| 2[263] | 3[247] | CGGGCCTGTAAGCCACACGGTCAGGATACCGG |
| 2[351] | 3[343] | TTTTTAGTACGGCTGCCCGTCAGG |
| 3[152] | 5[151] | CCATAATAATGGCCTCATCCACACGGTAAGGT |
| 3[168] | 2[165] | GTCAATCATTTCGCCTCCC |
| 3[184] | 5[183] | TCCTCACCCTGCACCATTCCGGCGGTGGCATG |
| 3[200] | 1[199] | ATCCCCATGCCATCACTTCAGCATAGGTTTTA |
| 3[216] | 5[215] | TTCGTAGCAGTATCCCAGCAGATAGATGCCCC |
| 3[232] | 1[231] | TGGGAGTCGAACGGGCGCTGAATATTCATCCA |
| 3[248] | 2[264] | CGGTCAGGTCGTGGTCCGTCATCAGATAACTG |
| 3[280] | 5[279] | TAATGCCACTTCTCCTCCTCTGTTCGCCCAGG |
| 3[296] | 1[295] | AGTGCCTCCACCGGCAGCACGGCGCGGATCGC |
| 3[312] | 5[311] | TCAGGCTGTATGCGGGAGATTACGGGCGCTCC |
| 3[328] | 1[327] | CACCGCATAGCGCCCGCGTCCGGCTAATGCCG |
| 3[344] | 5[343] | CTGCTGATCATCAGCGGCTCATTAAAAATTTC |
| 3[360] | 13[151] | GGCCTGCAAGCGCACAAAATCGCCACCTCTGACTTGAG |
| 4[263] | 5[247] | CAGCCCGGCGTTCATACACAATGGCCCATGTT |
| 4[383] | 5[375] | TTTTTGAAAACCCGCGGCACTCTT |
| 5[120] | 7[119] | TTTCGCGGGTTCATCCCCTTCCGGTGTTTTCT |
| 5[136] | 4[133] | CCGATTTCGCAGCAGCGTC |
| 5[152] | 7[151] | AAACCCCGGTTATTCGGGAAGTGACCTGCAGG |
| 5[168] | 3[167] | TCGCTTACGGTATAGGTTTTATTGATCTGCCG |
| 5[184] | 7[183] | CTGGCCACTGCTGCGCTTCGGTCAAGCTGCCA |
| 5[200] | 3[199] | CGTAGACGCGGGATATCGACATTTCGGCCCCC |
| 5[216] | 7[215] | TTTAATGGCCCATTTTTCGACCTGCGCCAGCG |
| 5[232] | 3[231] | CGGAACAGTCGGGTCAATCCCGCCGGTCCAGC |
| 5[248] | 4[264] | TTTTCGAGATTCAAATGCTGCAGCGATTGTCG |
| 5[280] | 7[279] | CGGGCGGTACGCGGCGAACGATGGGCCTGACG |
| 5[296] | 3[295] | GGCTGCACCAGCTCTGCTGTCACTGCGTCGCG |
| 5[312] | 7[311] | ATCGTTTCAACTGAAGCGCGCTTTCATAAAGT |
| 5[328] | 3[327] | GCTGGGCAAAGGCCATGCTGGCAATCGCCGGA |
| 5[344] | 7[343] | CTGATAGTGCAGTTCCCCCAGGCACCCGTGCC |
| 5[360] | 3[359] | CGTTTTGCCATAGGCGGGTTCAAGCCCAAGCT |
| 5[376] | 7[375] | TCTCGTAGTCGATGGCCATACGACTTCCGGGA |
| 5[392] | 15[119] | TCCGGCTTCTATCAGCATCGGCGATAAGTCGTGTCTTA |
| 6[263] | 7[247] | AGCCAGCAGCTGTCCCACAGTATTCATTGCGT |
| 6[415] | 7[407] | TTTTTACCGCTTCCTGCCCGGCAG |
| 7[88] | 9[87] | TAAGGCACCGGGCAGCGGCAAGTTACGGGTAA |
| 7[104] | 6[101] | TCTGCCAGCGTCAGTGTGA |
| 7[120] | 9[119] | TGTTGGTTGTTTACCTGTCATCAGCACTTTCA |
| 7[136] | 5[135] | ATCCTCTTACGGCACCGGGAAGCGTATCCGTA |
| 7[152] | 9[151] | CTCGCCAGCCTTCGTCCGTCTTTCTGCCCTCC |
| 7[168] | 5[167] | TGAGATCCGATCAAAAATATCCGGTTTTTGTT |
| 7[184] | 9[183] | GCGGGAAAAGGTCAGACACGGAAGACCGGCAT |
| 7[200] | 5[199] | CGCAGCGCCTCTTCAGCAGTCAGCCGGCTTTC |
| 7[216] | 9[215] | CATAAACGTCTGCACTTCCAGCTCCCGGCCCC |
| 7[232] | 5[231] | GAGTGCCTTTTTTCCTGCCATCCAGGATCACC |
| 7[248] | 6[264] | CGCTTTTTCAGAAACATCTGGCTCCCTCTTCC |
| 7[280] | 9[279] | GGATGCGAGTTCGCGCCCAGAATACTCAATGG |
| 7[296] | 5[295] | CGCCGCCCTGAAGGTAACGTCACCTTAAGACC |
| 7[312] | 9[311] | ACGCCCACCAGCCGGTCAGCCTTTTGGCGCTC |
| 7[328] | 5[327] | CGCACTGGCTGCGGGCTTCCTGAAACGGACCT |
| 7[344] | 9[343] | GTGGAGTACTGCGGCGTAATACGCTCGAGCAT |
| 7[360] | 5[359] | GGGCGTAACGGAGCCTATCCAGTCCGTCACCG |
| 7[376] | 9[375] | AAGCTGCTCGGTACTTTTGCGCCTAAACACAT |
| 7[392] | 5[391] | ACACCCAGAACTTCTTTCAGACCAGTACTCAG |
| 7[408] | 9[407] | CGATATACTGCAGGTTCAGTGAGTCCACGGGT |
| 7[424] | 17[87] | TGACTGCTCAAACACGGAGAAGATCCTTTTTGATAATC |
| 8[263] | 9[247] | CGCTGACTTGCAGGTCCCCTGCGTTTCATACA |
| 8[447] | 9[439] | TTTTTTAGCCGTTATCCGCCCGCC |
| 9[56] | 10[37] | CAGATCGTCCTGATGCAGCTGGATGGCGTTGGCGG |
| 9[72] | 8[69] | GCATTGCCCTTCCTGTCGC |
| 9[88] | 10[80] | AGTTGGGCACTGAGCCGGAAAAAA |
| 9[104] | 7[103] | TGCATCCAGTCATGCAGTGCCGCAGGGCGTAA |
| 9[120] | 10[112] | CTCGGTGGCCGATGCCCAGATAGC |
| 9[136] | 7[135] | ACCGCAACTGTACTGTTGCCACCCGCTGCACC |
| 9[152] | 10[144] | AAATCCGCCCTCGCGGGAAAAGGC |
| 9[168] | 7[167] | CCGTGATATGGCCGTAAACTCCACCAGCGCAC |
| 9[184] | 10[176] | ATTCGCGCATCCTCGGCAAACTCT |
| 9[200] | 7[199] | CATGCCGTTGCAATATATTTTTTCTACTGATG |
| 9[216] | 10[208] | AGAAGGGTCGTTTTCGCTCAACGT |
| 9[232] | 7[231] | TGGGCGTTCGCTGTGTCATGCCGGAAGCAGTC |
| 9[248] | 8[264] | TAAAATCCGCGTATCCAGCTCACTAAATCCAT |
| 9[280] | 10[272] | TGGCGGCAAACGAACAGTTCACCG |
| 9[296] | 7[295] | CTTCACAACCCGCTGCTCCTGACTCGAATTTT |
| 9[312] | 10[304] | TGCAGCTGAGCCGCGACGAACTGG |
| 9[328] | 7[327] | GCAGCGTGGGCAATTTCACCAATCGACTCGTT |
| 9[344] | 10[336] | CTTCATCTTCGGGCTGACCATCCG |
| 9[360] | 7[359] | ACGCTGTACCCAGCCGGACCGGCGGCTCATCT |
| 9[376] | 10[368] | TTGCACCGGTCGCCGGTATTGTTC |
| 9[392] | 7[391] | CCCGTCCTCACCCGGCATCAGGTGCATACGAG |
| 9[408] | 10[400] | TCAAAAACTTAATCTGCACACCGG |
| 9[424] | 7[423] | ACGAGGCGCGTATCCTGAGCCGTCCGCAGCAG |
| 9[440] | 10[432] | GGGTAACTTGACGTAATATCCCAG |
| 9[456] | 19[55] | ATCCATGTCCATTTCTGCGCACCACGATGCCTGTAGCA |
| 10[79] | 9[71] | GACCCGACGATATGATCTGCGCGG |
| 10[111] | 9[103] | GCCAGCTTGGGCGATGAACAGGGC |
| 10[143] | 9[135] | ACGGGCTTCTTCCTCCGTTCCACG |
| 10[175] | 9[167] | TTCCATGCCGCTTCAATGCCACCG |
| 10[207] | 9[199] | CAATGCAGCAGCAGTCAGCGATGT |
| 10[239] | 9[231] | CATCATGGTAAACGTGGGGAATGG |
| 10[271] | 10[240] | TTAAAGGCGTGCATGGCCACACCTTCCCGAAT |
| 10[303] | 9[295] | TATCCCAGGTGGCCTGTACATCGC |
| 10[335] | 9[327] | GAACTGTGTCCGGAAACGTGTTCT |
| 10[367] | 9[359] | GGGTTGCTGATGCGCTGCTCCATC |
| 10[399] | 9[391] | CACGGCAGTTCCGGCTCGAGTCTG |
| 10[431] | 9[423] | CGCCGCACCGCTGTCAGTGAATGA |
| 10[474] | 9[455] | GCATCCAGCCAGGATACCCGTCCTCGCCACGGGGT |
| 12[263] | 11[247] | CATTCCTCCCGGGCGGTCTGCGTGCGCCGATG |
| 11[184] | 13[183] | GATTTCCATGTAGGGGTTGCCATCGGGGGGCG |
| 11[200] | 11[183] | TTCTCCAGCTGACCTGCACTGCAGTTCATGTCGTTGGCAAATCAGCGT |
| 11[216] | 13[215] | CGTAATTACAGTGTCTCCCGGACGCGCGGCCT |
| 11[232] | 11[215] | CATCATGAACCAGACGCCGGGAGGCGGCACTGGCAAGCAACGCAGCAC |
| 11[248] | 12[264] | GAGCCTGTTCATAGGCCTTCCTGAGGCCAGCT |
| 11[280] | 13[279] | CACTCAACATCGAGTAGCGGGCGAGTGGTTAT |
| 11[296] | 11[279] | CAGTGCTGCTGGCCAGCTCCTTTTCATCGGGCTTGTTCGGGGAGGAAC |
| 11[312] | 13[311] | GACGTCCCTCCCAAGCTCGTATGTCGGCTGAC |
| 11[328] | 11[311] | TGGCCGATTTACATAGCCTCCTTCATAGCTCTGCCGTCTTGAGCGGGA |
| 11[336] | 21[175] | TAAGCGAGAAACTGGGAAGTCC |
| 14[263] | 13[247] | AGGTTCTCAACATCTGGCGGGTTGGGACACCT |
| 14[351] | 13[343] | TTTTTGCTGGGCCTCCCAAGCGTG |
| 13[152] | 15[151] | CGTCGATTCTGGTATCTTTATAGTTACCGGAT |
| 13[168] | 14[165] | GCTCGTCACACCTTATGGC |
| 13[184] | 15[183] | GAGCCTATGAGCGCACGAGGGAGCGGGGGTTC |
| 13[200] | 11[199] | GCCAGCAATCATCCGGAAGATGGCCACCCTGT |
| 13[216] | 15[215] | TTTTACGGGGTATCCGGTAAGCGGAACGACCT |
| 13[232] | 11[231] | CTTTTGCTCGTCCATCCGGGACTGCTGTGAGC |
| 13[248] | 14[264] | TCTGCGCAGCCGGTTTCCCAGGCTCTCTCTCA |
| 13[280] | 15[279] | TTCCTCAGGCGAAGTTTATTCCGATCGAAGTT |
| 13[296] | 11[295] | TTCTCCTTGACCTTCGCATCTTCCGAGGTTGC |
| 13[312] | 15[311] | CGAAGACGGAATCTTCGGCTCGCTGTCCGGGT |
| 13[328] | 11[327] | AACGGCCTATCGGAAGGAACTCCTAGAGGGAC |
| 13[344] | 15[343] | TATGTGGGCACTTCGACGGCAAACAGCGCTTT |
| 13[360] | 23[151] | TCGTCCGTCTTATCACAGGCTTCCCCAGGCCGTTACGC |
| 16[263] | 15[247] | CAGCGCTTCGCCACGCTTCCCGAAGTGAGCTA |
| 16[383] | 15[375] | TTTTTATAGAGATCGAACGTCAAC |
| 15[120] | 17[119] | CCGGGTTGATCCTGTTACCAGTGGAGTTTTCG |
| 15[136] | 16[133] | ACGATAGTCCTGTCGGGTT |
| 15[152] | 17[151] | AAGGCGCACTGTAGCACCGCCTACGAAAAGAT |
| 15[168] | 13[167] | GCTGAACGTTCCAGGGGGAAACGCTTTGTGAT |
| 15[184] | 17[183] | GTGCACACGTAGCCGTAGTTAGGCTTTTCTGC |
| 15[200] | 13[199] | TTGGAGCGCAGGGTCGGAACAGGAGGAAAAAC |
| 15[216] | 17[215] | ACACCGAAAGAGCGCAGATACCAAAAAAACCA |
| 15[232] | 13[231] | CCTACAGCGGGAGAAAGGCGGACATTCCTGGC |
| 15[248] | 16[264] | TGAGAAAGTTTCAGATAGGCGAAGGCTTTTCA |
| 15[280] | 17[279] | GTCGCCGTGCACTAATCCCTTGTCACTCGTAG |
| 15[296] | 13[295] | ATGAGAACTCCATCCCTTCCGAGGCGTAAACC |
| 15[312] | 17[311] | CTTTCTCCGAGCATTTTCAGCTCCCGTATCTT |
| 15[328] | 13[327] | ACGGAGGACTGTCGCCCATCCTCTGCTTCATA |
| 15[344] | 17[343] | ATCATCTCCCCTCATGGTAGAGAGCGGATGAG |
| 15[360] | 13[359] | TCGAGACGAGTGTATCCGTCCCTTCAGGTTTA |
| 15[376] | 17[375] | GTAGGGGATCATAAGGATTGGCCCCTGGTCTC |
| 15[392] | 25[119] | TTCTTCCAAGTTATCACCCCAATCTCAAGTCCGCGCGT |
| 18[263] | 17[247] | AGCGCTTGCAACTCTTTTTCCGAACCGGATCA |
| 18[415] | 17[407] | TTTTTTGGCCCCTTCCTCAACCCG |
| 17[88] | 19[87] | TCATGACCCATTTTTAATTTAAAATTAACTGG |
| 17[104] | 18[101] | TTAACGTGCTGCTGCCAGT |
| 17[120] | 19[119] | TTCCACTGACTCATATATACTTTAGCAACAAT |
| 17[136] | 15[135] | ACCCCGTAATACCTCGCTCTGCTAGACTCAAG |
| 17[152] | 19[151] | CAAAGGATGATTAAGCATTGGTAAAAGTTGCA |
| 17[168] | 15[167] | GATCCTTTCACCACTTCAAGAACTGCGGTCGG |
| 17[184] | 19[183] | GCGTAATCAATAGACAGATCGCTGCCGGCTGG |
| 17[200] | 15[199] | GCAAACAAATACTGTCCTTCTAGTAGCCCAGC |
| 17[216] | 19[215] | CCGCTACCCGACGGGGAGTCAGGCAGCCGGTG |
| 17[232] | 15[231] | TTTGTTTGGGTAACTGGCTTCAGCCTGAGATA |
| 17[248] | 18[264] | AGAGCTACGCGAAGGGTATGTCGTTATGAGGT |
| 17[280] | 19[279] | ATGTCAATAGGATCATGCTGCCGCAATGTGCG |
| 17[296] | 15[295] | GATGCTCTTCGTCGCCTTCCATTGTGTAGGTT |
| 17[312] | 19[311] | GTCCCTTAGCTTTCCATCCTCGGTCTAAATAC |
| 17[328] | 15[327] | ACGTCCTGTCGCAATCGCGAAGGCTTCACAAC |
| 17[344] | 19[343] | TAAAGTAGGCCGTTTTCCTTCTTGGACAATAA |
| 17[360] | 15[359] | GACCTCAACTCGGCGAACTCCTCGCCTCTCCG |
| 17[376] | 19[375] | CCGAGGAATCAAAAGTCCGGTCGTTGAAAAAG |
| 17[392] | 15[391] | GAACCTTTTCGTCGGCGTAGCTTAGATCCACG |
| 17[408] | 19[407] | CTTAACCGAATCGTCCTTCAGGAGCCGTGTCG |
| 17[424] | 27[87] | GTCCCGTGCCTCTCGGCGGGCTTCGTCGGGTCGAACTC |
| 20[263] | 19[247] | AGGCCGCTTGGTAAGCCCTCCCGTCAGCACTG |
| 20[447] | 19[439] | TTTTTTTATCTTCTTGTTTGCCTT |
| 19[56] | 22[37] | ATGGCAACTGAAGCCATACCAAACGACGAGCGTGA |
| 19[72] | 20[69] | GCAAACTAGGATCTAGGTG |
| 19[88] | 22[80] | CGAACTACACTCGCCTTGATCGTT |
| 19[104] | 17[103] | GCTTCCCGGATTGATTTAAAACTTAAAATCCC |
| 19[120] | 22[112] | TAATAGACCCGCTTTTTTGCACAA |
| 19[136] | 17[135] | GGCGGATACTGTCAGACCAAGTTTAGCGTCAG |
| 19[152] | 22[144] | GGACCACTTCTGACAACGATCGGA |
| 19[168] | 17[167] | CGGCCCTTAGATAGGTGCCTCACTCTTCTTGA |
| 19[184] | 22[176] | CTGGTTTAACCATGAGTGATAACA |
| 19[200] | 17[199] | AAATCTGGAACTATGGATGAACGATGCTGCTT |
| 19[216] | 22[208] | AGCGTGGGTGACAGTAAGAGAATT |
| 19[232] | 17[231] | TATCATTGATCGTAGTTATCTACAAGCGGTGG |
| 19[248] | 20[264] | GGGCCAGATGGATCCGTACGGGGAGCGGCACC |
| 19[280] | 22[272] | CGGAACCCCACTATTCTCAGAATG |
| 19[296] | 17[295] | TTATTTTTTATGTAGTCAGTGTCGAACTGCTG |
| 19[312] | 22[304] | ATTCAAATACGCCGGGCAAGAGCA |
| 19[328] | 17[327] | GCTCATGAAAAATTCTTATGACAGTCGCTGGG |
| 19[344] | 22[336] | CCCTGATATCTGCTATGTGGCGCG |
| 19[360] | 17[359] | AATAATATACTCAATCTTAAACTCAGTTTCCA |
| 19[376] | 22[368] | GAAGAGTAGAACGTTTTCCAATGA |
| 19[392] | 17[391] | CAACATTTGGCGTAGAAGTAGGGTCTTCTTCT |
| 19[408] | 22[400] | CCCTTATTGCGGTAAGATCCTTGA |
| 19[424] | 17[423] | TGCGGCATACTTCCTCAATGGCAGTTACAACC |
| 19[440] | 22[432] | CCTGTTTTACGAGTGGGTTACATC |
| 19[456] | 29[55] | CAGAAACGCTGGTGAAAGTTGACTGAAATGCCTCAAAA |
| 22[79] | 19[71] | GGGAACCGGAGCTGAAAACGTTGC |
| 22[111] | 19[103] | CATGGGGGATCATGTATTACTCTA |
| 22[143] | 19[135] | GGACCGAAGGAGCTAATGGATGGA |
| 22[175] | 19[167] | CTGCGGCCAACTTACTTCTGCGCT |
| 22[207] | 19[199] | ATGCAGTGCTGCCATATTGCTGAT |
| 22[239] | 19[231] | CATCTTACGGATGGCATCTCGCGG |
| 22[271] | 22[240] | ACTTGGTTGAGTACTCACCAGTCACAGAAAAG |
| 22[303] | 19[295] | ACTCGGTCGCCGCATACTATTTGT |
| 22[335] | 19[327] | GTATTATCCCGTATTGATGTATCC |
| 22[367] | 19[359] | TGAGCACTTTTAAAGTAATGCTTC |
| 22[399] | 19[391] | GAGTTTTCGCCCCGAATGAGTATT |
| 22[431] | 19[423] | GAACTGGATCTCAACACCCTTTTT |
| 22[474] | 19[455] | AAAAGATGCTGAAGATCAGTTGGGTGCTGCTCACC |
| 24[263] | 21[247] | GCTGGCGGGTGGGTGATGATGATTTATCCGGC |
| 21[184] | 23[183] | GCGGTGGCGGTCTCCAAGCAGGCTAGCGCGCC |
| 21[200] | 21[183] | GGGGCAACTATGTTCTCCCACAACCCTCTCTCGGGCTCTTTTGCAGAA |
| 21[216] | 23[215] | CCTTGCATCTTCTTCTTTATCTTCTAGCTGTT |
| 21[232] | 21[215] | GTTGAGCGGAGGGGTACAGGGATCTAAAATCTAGGTACACGTCATATT |
| 21[248] | 24[264] | GAGACGTTATGACGCAGGCATTATCAGCTCCT |
| 21[280] | 23[279] | AGATAAAAGGGGATTCACTGGCTGGGCGTGGA |
| 21[296] | 21[279] | AGGGGTCATTTTCGCAGATATAACGGGCATCAGTAAAGTCGCTCGCAG |
| 21[312] | 23[311] | CCATTTGAGCGGGCAGGCAACATGCGAGCCTT |
| 21[328] | 21[311] | GGCGTCTCAGAGAATGCCATCACGGGTCCAGATCCCGGTCTCCGGCGT |
| 21[336] | 1[175] | TTTGTCGCCAAACGTCTCACCG |
| 26[263] | 23[247] | ATCTGACATTCCTCTCGATCGGGTCCTGTAAT |
| 26[351] | 23[343] | TTTTTATTTAAGATACTCGGAGAG |
| 23[152] | 25[151] | TCTCTGCATCCTTGATGGTCATCGCACCGCAT |
| 23[168] | 26[165] | GCAGTACCCGGGATAAATC |
| 23[184] | 25[183] | CTTGCATAAGATTACCTTAAAGCCCGCGTTTG |
| 23[200] | 21[199] | AACCGTAGTGCCTCTCCTCTAGGACGACCTGT |
| 23[216] | 25[215] | TGCCAGGATATTGTGGCAAAAAATAGCGCGCC |
| 23[232] | 21[231] | CGCCTCTGCAATCGTGGCCTTCATCCTTCTCT |
| 23[248] | 26[264] | CGAGGAGCGGTGCCTCTCACTTTTCACGCTCA |
| 23[280] | 25[279] | GCCACGGACGCTTCATGGTGAGCGGCCAGCGC |
| 23[296] | 21[295] | GGCCAGACAAAACGCATAAAATGCCACGCTGG |
| 23[312] | 25[311] | CAATACGCCCAGGCACCAGAAGCCAGTCGGCT |
| 23[328] | 21[327] | CAGGAACGTGCTCCTCCCCGCAATGGCCAAAC |
| 23[344] | 25[343] | CCTTCCTGCTTTTCACGGTAGTTTCGGCATCA |
| 23[360] | 3[151] | CATCATCAAAAGCAGCAAGTGTTCATCGTCGTGGCGGC |
| 28[263] | 25[247] | CGGTGCTTTTCTTTTTGACGGTTTGAGGAACT |
| 28[383] | 25[375] | TTTTTTTCATCATAACGTGTTGCG |
| 25[120] | 27[119] | TGTTATCTTCGCTATCTCGCTCCATATCCTCC |
| 25[136] | 28[133] | TCGTCTATTTATGTACTCC |
| 25[152] | 27[151] | ACTTCTTCCAAAGCTTCAAGAACCTTATCACC |
| 25[168] | 23[167] | GAAGAAGCGTACTTTTCCTCTATCCACTCCTT |
| 25[184] | 27[183] | TAGAAGCCCTCACGGCCTTCTCGACTCGCGGC |
| 25[200] | 23[199] | CGAGCTCACCGTCGGTGTCGCTGTGCCGTAGT |
| 25[216] | 27[215] | CGGAAGTTTGCTCAGCTTTTCGGTGGGACCGG |
| 25[232] | 23[231] | ATATACTTCAGCATCGGCTCCAGGTCTTGATG |
| 25[248] | 28[264] | CCATAGCCTTTGCCATACCACGGGTGTTATCC |
| 25[280] | 27[279] | CAGCAGCGATGCGGATTCTTTCGGCGGTCTGT |
| 25[296] | 23[295] | TCACGAATACGCCCATTAGTGAAATGGACTTT |
| 25[312] | 27[311] | CAACGTGGAAAGGGCAGTGTTTCCAGCCCGGC |
| 25[328] | 23[327] | AAAGTTCTTTTGCCGCTTTACCGCTTGTCACC |
| 25[344] | 27[343] | CCATCCGTTCGCTGCCCATCGCATAACCTGAC |
| 25[360] | 23[359] | AGATAAGGCCGCCACATCCACCGATTCAATAT |
| 25[376] | 27[375] | CTGCTTATCAGACTTCACCACATTCCCGGTAT |
| 25[392] | 5[119] | AAGTAGGCATAAACACCCAAGCGGTTATAAATCTGCTC |
| 30[263] | 27[247] | AATTAGCAGATCACCAGCTTCTCCTCGTTATC |
| 30[415] | 27[407] | TTTTTGCAGCATTTTGGTTCATGG |
| 27[88] | 29[87] | GTCGAACGCAATGTAGTACTCGGCATATCAAC |
| 27[104] | 30[101] | CTGTCGCCGTCACGCCTCA |
| 27[120] | 29[119] | CAGAGCCCAATTCTCTCAACGGCTCTCCATTT |
| 27[136] | 25[135] | GATGTAGCCTCGCCTGCGTCTCTTTGCCTTCC |
| 27[152] | 29[151] | GTTCCAGGAGGTCTTCCTTGCGGTTCGCCAAG |
| 27[168] | 25[167] | TGACTCCTCGTCACCGTCCTTTAGTTCGTGAC |
| 27[184] | 29[183] | CAACCTCTTCAAACCAACCTGTCTAAACGCCC |
| 27[200] | 25[199] | GCAACGTGAACTTCTTTGACTATCCTCGTACT |
| 27[216] | 29[215] | TTGCCTTGGACTCAAGTTCCCTTCAATCCAGA |
| 27[232] | 25[231] | TAAATCCCGGCGGAACCTCGTACTTGGCGTTG |
| 27[248] | 30[264] | TGTATGTGTCCGCCCATTCAACGGGAGATAGT |
| 27[280] | 29[279] | GGCTCCGGGGCCTGCGCACGCGTACTGGAGAT |
| 27[296] | 25[295] | GTGAGCGACAGCGCCCTTCCTGGTACGGAATA |
| 27[312] | 29[311] | GCGGACAAGAGTCTCTGGCATTCTCTTCAACC |
| 27[328] | 25[327] | AGCCTGTTTCATGATGGCCCGCTGGTTTTCAT |
| 27[344] | 29[343] | TGTTCGATAAGTACAGAATGCGGTATTTCAGC |
| 27[360] | 25[359] | TCAGCAACCACCTCACGGATGTAGCGGCAACC |
| 27[376] | 29[375] | CAGTTCATGGCAATTTCACCTGCGGACGGCGG |
| 27[392] | 25[391] | GCTGCTTTGAATAACCGACACGGGGCTCTATA |
| 27[408] | 29[407] | CTTTGATGTGCACCGACAGGGGGACCTTGCCA |
| 27[424] | 7[87] | TTTCAGGAAATCAACATTTGTCATTCATCCTCTCCGGA |
| 32[263] | 29[247] | GCGTTCGTCAAGCTCAGCTAATTATCTATCAA |
| 32[447] | 29[439] | TTTTTCGGTTTTCCAGTGTTCCTG |
| 29[56] | 34[37] | TGTTCTTTGGTCTGGTTATAGGTACATTGAGCAAC |
| 29[72] | 32[69] | ATTGGGATGTCGTACTTGT |
| 29[88] | 34[80] | GGTGGTATGTCTTTAAAAAGGCCG |
| 29[104] | 27[103] | ATTTTTTTGGGAGAACCTGGTTCTGTATCGCC |
| 29[120] | 34[112] | TAGCTTCCCCGGATAAAACTTGTG |
| 29[136] | 27[135] | TGAAAATCAACCGAAGGCTCTCAGTTGAGCAC |
| 29[152] | 34[144] | CTAGCTTGATTCATCAGGCGGGCA |
| 29[168] | 27[167] | CCAATAAACGTCTTCTGGTAGCGCGCGTATTT |
| 29[184] | 34[176] | GGCGGCAATCTTTCATTGCCATAC |
| 29[200] | 27[199] | TCTGAACAGGCTTCAGCCAAGCACTGGCAACG |
| 29[216] | 34[208] | TGGAGTTCGGTGAACACTATCCCA |
| 29[232] | 27[231] | TTACTGGAAGCTTGGCTGCAGGTCTAGTCCTT |
| 29[248] | 32[264] | CAGGAGTCACTCAATAGTTCCTGGAGTCGATG |
| 29[280] | 34[272] | CTGCCTCGTCATAAATAGCGAAAA |
| 29[296] | 27[295] | CCGCAGTTTCAGTTCCTGTGCGTCCCTGAACA |
| 29[312] | 34[304] | TCCCGGCGGAAGAACGGAAACGCC |
| 29[328] | 27[327] | CGTTCTCATTCCACCACTTCAGCGAATGCCGC |
| 29[344] | 34[336] | ATCCCTTTTATTTTAAATAAAAAC |
| 29[360] | 27[359] | CATTTTATATCCGCGACAGCACGAATATTCAC |
| 29[376] | 34[368] | CAGAGTCACAGCACCTGTCGTTTC |
| 29[392] | 27[391] | CTCATTACGCCCGTCGAGAATACTCCAGCGCG |
| 29[408] | 34[400] | CCGCCTCGAGGAAACGACAGAGGC |
| 29[424] | 27[423] | GCATTCCCTTCCGGAAAACGCCGCATATCCCG |
| 29[440] | 34[432] | CCAGTTCTCCATTGTTCATTCCAC |
| 29[456] | 9[55] | CGGATACTCGCACCGAAAACATAGCCGTTATTGCGTAC |
| 34[79] | 29[71] | TAATATCCAGCTGAACACGATGCC |
| 34[111] | 29[103] | CTTATTTTTCTTTACGATCCAGTG |
| 34[143] | 29[135] | AGAATGTGAATAAAGGTTAGCTCC |
| 34[175] | 29[167] | GAAATTCCGGATGAGCGATTCTCA |
| 34[207] | 29[199] | TATCACCAGCTCACCGCCGAGCGT |
| 34[239] | 29[231] | AAAACGGTGTAACAAGTGAGGTCA |
| 34[271] | 34[240] | CCCGAAATCGGCAAAATTCAGTTTGCTCATGG |
| 34[303] | 29[295] | TTAAACCGGAAAATTTCTGGCCTG |
| 34[335] | 29[327] | ATTAAGTTATGACGAACAGCTTTT |
| 34[367] | 29[359] | CTTTCTTTTCAGAGGGCGGCATAC |
| 34[399] | 29[391] | CAAAAAGCCTCGCTTTTAAAGCAC |
| 34[431] | 29[423] | GGACAAAAACAGAGAACAGAACGG |
| 34[474] | 29[455] | TGTCAGCCAGCTGCTTTTTGTTGACTTGAATGGTA |

Table S5. Sequences of Cy5-modified staple strands.

| Sequencing Primer Name | Sequencing Primer Sequence |
| --- | --- |

| large triangular DNA origami 1 | GACCCGACGATATGATCTGCGCGG |
| --- | --- |
| large triangular DNA origami 2 | GGGAACCGGAGCTGAAAACGTTGC |
| large triangular DNA origami 3 | TAATATCCAGCTGAACACGATGCC |
| large triangular DNA origami 4 | GGGTAACTTGACGTAATATCCCAG |
| large triangular DNA origami 5 | CCTGTTTTACGAGTGGGTTACATC |
| large triangular DNA origami 6 | CCAGTTCTCCATTGTTCATTCCAC |
| conventional triangular origami 1 | ACCAACCTAAAAAATCAACGTAACAAATAAATTGGGCTTGAGA |
| conventional triangular origami 2 | AAAGAAGTTTTGCCAGCATAAATATTCATTGACTCAACATGTT |
| conventional triangular origami 3 | TATCTTACCGAAGCCCAAACGCAATAATAACGAAAATCACCAG |
| conventional triangular origami 4 | ACGACAATAAATCCCGACTTGCGGGAGATCCTGAATCTTACCA |
| conventional triangular origami 5 | GCCACCGAGTAAAAGAACATCACTTGCCTGAGCGCCATTAAAA |
| conventional triangular origami 6 | AACTCACATTATTGAGTGTTGTTCCAGAAACCGTCTATCAGGG |
